# The optimized algorithm based on machine learning for inverse kinematics of two painting robots with non-spherical wrist

**DOI:** 10.1371/journal.pone.0230790

**Published:** 2020-04-03

**Authors:** Xiaoqi Wang, Jianfu Cao, Lerui Chen, Heyu Hu

**Affiliations:** Faculty of Electronic and Information Engineering, Xi’an Jiaotong University, Xi'an, Shaanxi, China; Nanyang Technological University, SINGAPORE

## Abstract

This paper studies the inverse kinematics of two non-spherical wrist configurations of painting robot. The simplest analytical solution of orthogonal wrist configuration is deduced in this paper for the first time. For the oblique wrist configuration, there is no analytical solution for the configuration. So it is necessary to solve by general method, which cannot achieve high precision and high speed as analytic solution. Two general methods are optimized in this paper. Firstly, the elimination method is optimized to reduce the solving speed to 20% of the original one, and the completeness of the method is supplemented. Based on the Gauss damped least squares method, a new optimization method is proposed to improve the solving speed. The enhanced step length coefficient is introduced to conduct studies with the machine learning correlation method. It has been proved that, on the basis of ensuring the stability of motion, the number of iterations can be effectively reduced and the average number of iterations can be less than 5 times, which can effectively improve the speed of solution. In the simulation and experimental environment, it is verified.

## Introduction

For the 6R painting robot, non-spherical wrists are usually adopted because of operation requirements. It is mainly divided into orthogonal non-spherical wrist and oblique non-spherical wrist. For the orthogonal non-spherical wrist configuration, three adjacent axes are parallel to each other to meet Pieper criterion, there is an analytical solution, and the simplest analytical solution is given in this paper. For the oblique non-spherical wrist configuration, the Pieper criterion is not satisfied and there is no analytical solution. Among many methods, the elimination method has the most complex calculation process, but it has the highest accuracy and can get all the solutions under the same terminal position. In this paper, the elimination method was optimized to improve the speed of solution and to supplement the integrity of the method. Gaussian damped iteration method is derived by the most widely used damped least squares method. The Gauss damped iteration method is an efficient and stable method. It has the advantages of fast convergence of Newton iteration method, smooth motion of damping method and less error after optimizing damping factor. But in the embedded environment, it cannot achieve the same speed as the analytic solution of general configuration. On the basis of the algorithm, this paper introduced the enhanced step length coefficient, analyzed the affecting factors of the coefficient, studied the coefficient through machine learning classification and regression methods, and compared the performance of various models. After optimization, the average number of iterations was less than 4 times, which effectively improved the solving speed. Next, we will introduce robotic products using two configurations, some general new methods and optimization methods in other papers, and machine learning methods for studying the enhanced step length coefficient.

Both configurations are common in robot products. For the orthogonal non-spherical wrist, Yaskawa's EPX series with Lemma wrist, Fanuc's M-710iC/50E, Kawasaki's K-series with BBR wrists, etc. For the oblique non-spherical wrist, Yaskawa's EPX and MPX series, Fanuc's P-50iB and P-250iB, Kawasaki's K Series with 3R wrist, Comau's NJ4 series with Hollow wrist, Staubli's TX250, etc.

[1] and [2] proposed an elimination method for inverse kinematics of 6R robots, which simplified the inverse kinematics of the robots into a 16-degree polynomial problem with one variable. Then the matrix eigenvalue decomposition was used to replace solving equation of higher degree. However, the elimination process is complex and time-consuming, and the final expression of the matrix is not derived, so the solution requires multiple steps. [3] proposed the pseudo-inverse Jacobian matrix method, which will lead to great joint velocity in singular configuration. Elimination method and Jacobian inverse iterations method were combined in [4] to obtain the full set of inverse kinematic solutions. In [5,6], a damping least squares method (DLS), also known as Levenberg-Marquardt stabilization method, was proposed, and the damping factor was selected based on the minimum singular value. In the singular configuration, the damping term was introduced to balance the error and joint velocity and make the motion more stable, which is an iterative algorithm with better comprehensive performance. The selection of damping factor directly affects algorithm performance. [7] proposed selective damped least squares method (SDLS), which is better than DLS in the selection of damping factors. [8] proposed the Gaussian damping method. Based on the Gaussian distribution of the damping factor, it is the method of determining damping factor with Gaussian function. This method can effectively ensure the continuity of joint velocity and avoid unnecessary errors. However, the average number of iterations of this method is large, and the solving speed is still not as fast as the analytical solution. A new numerical CCD method was proposed in [9], for any differentiable type of joint and demonstrated its use for serial-chain manipulators with coupled joints. The elimination method was optimized with geometric algebra and a new representation of the Euclidean motion group respectively in [10–11]. Four Jacobian-based methods of solving the inverse kinematics task have been improved and evaluated in [12]. Two methods (differential geometric method and variational method) of the extended Jacobian algorithm were examined in [13] to address the approximation of the Jacobian pseudo inverse. An implementation of the inverse kinematics solution for a robot based on Conformal Geometric Algebra was proposed in [14]. A double quaternion based kinematics formulation for the 6R robots was introduced in [15–16] to avoid kinematic singularities. The product of exponentials method based on screw theory was employed for kinematics modeling to avoid the problem of singularity. The inverse kinematics of the 6R robot manipulator was solved by adopting analytical, geometric, and algebraic methods combined with the Paden Kahan subproblem as well as matrix theory in [17–19]. Various optimized numerical and iterative methods failed to achieve the same performance as analytical solutions.

Various soft computing methods [20–26] based on artificial neural network and genetic algorithm are also the research hotspots in recent years. Three different soft computing methods were presented in [20]. Artificial neural network, adaptive neuro fuzzy inference system and genetic algorithm method were compared. Neural network and genetic algorithm were combined in [21]. A neural-network committee machine was designed in [22] to improve the precision of the solution. An intelligent algorithm based on extreme learning machine and sequential mutation genetic algorithm was proposed in [25]. An online adaptive strategy based on the Lyapunov stability theory was presented in [26]. Radial Basis Function (RBF) Neural Networks (NNs) was employed and Quadratic Programming (QP) method was incorporated in the training algorithm of the NNs. Various soft computing methods can achieve accuracy up to the micron level, but the solution speed is still a problem.

In this paper, the enhanced step length coefficient was studied by machine learning methods. Classification and regression methods commonly used in machine learning mainly include DecisionTree(DT) [27] and NaiveBayes(NB). DT is a prediction model to establish a mapping between object attributes and object values. It has fast speed, and is easy to visualize the model, but easy to overfitting. NB takes the maximum conditional probability based on Bayesian criterion. It needs few parameters to estimate and is insensitive to missing data. However, assuming that attributes are independent of each other, it will affect the accuracy. K-NearestNeighbor(KNN) [28] is based on the limit theorem. Decision-making depends on a very small number of the nearest neighbor samples. It is suitable for multi-classification problems. LogisticRegression(LR) is suitable for continuous and categorical independent variables and is widely used in medical related fields. It is sensitive to multiple collinearity of independent variables in the model. RandomForest(RF) [29] integrates the set of decision trees with control variance by using the idea of bagging. Gradient Boosting Decision Tree(GBDT) [30] generates a weak classifier in each iteration through multiple iterations. Each classifier trains on the basis of the residual of the previous one. Support Vector Machine(SVM) estimates regression based on a series of kernels. In this paper, the commonly used RBF nonlinear kernels are used. Bagging method constructs a series of predictive functions and combines them into a predictive function. ExtraTreeRegressor(ETR) [31] is similar to RF and consists of many decision trees. Each decision tree is obtained by using all training samples, and the bifurcation values are obtained completely randomly. XBGboost combines all the predictions of a group of weak learners to train a strong learner through additive training strategies.

## Materials and methods

### Inverse kinematics of orthogonal non-spherical wrist robot based on analytical method

**Fig 1:**
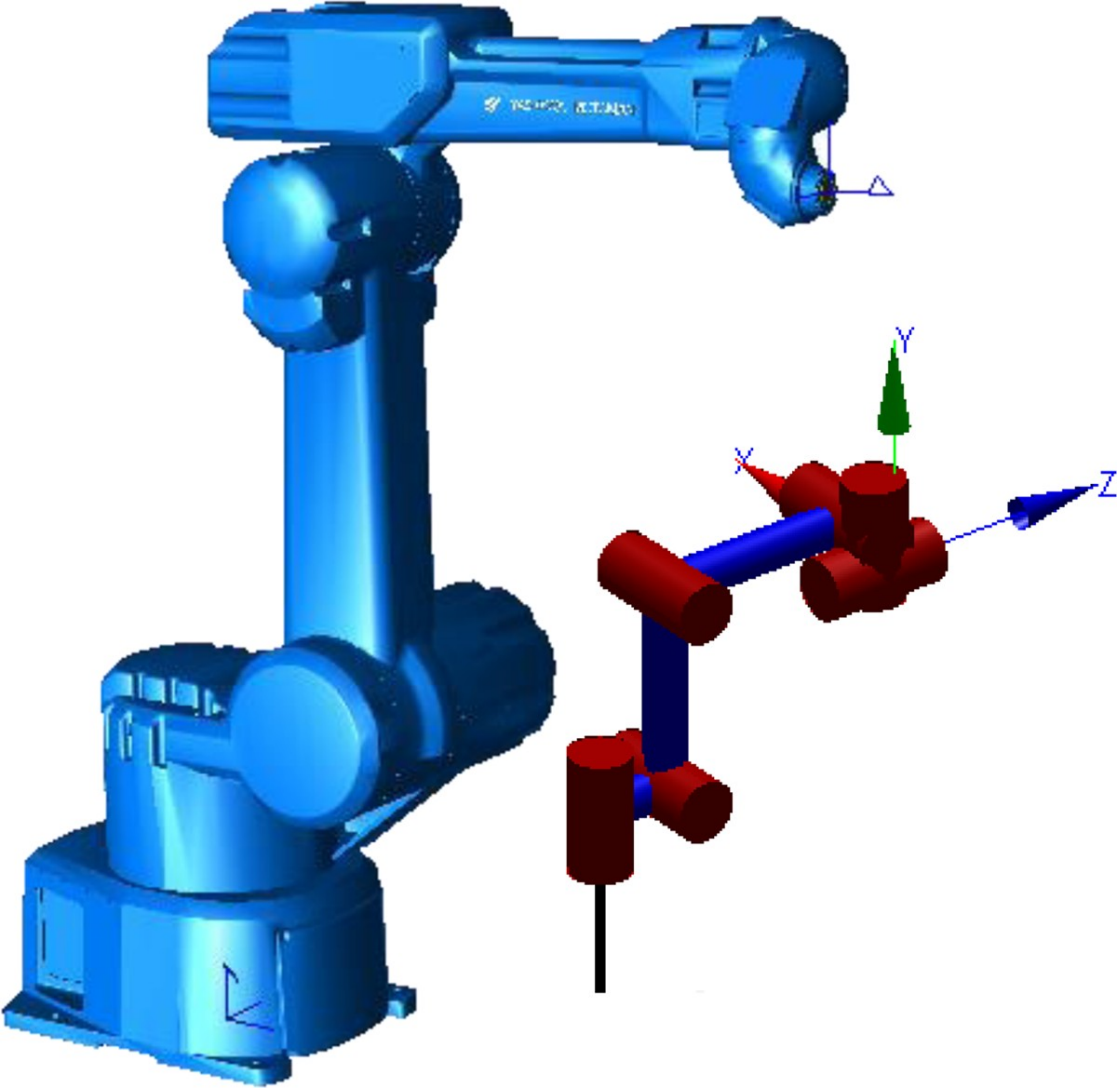
Model of orthogonal non-spherical wrist robot.

YASKAWA-EPX2050 (lemma wrist) was taken as an example in this paper. The model is shown in Fig 1, and DH parameters is shown in Table 1.

**Table 1:** DH parameters of orthogonal non-spherical wrist robot.

	$a_{i-1}\left(m\right)$	$\alpha_{i-1}{(}^{\circ })$	$d_{i}\left(m\right)$	$\theta_{i}{(}^{\circ })$
1(S)	*a*_1_	90	0	*θ*_1_
2(L)	*a*_2_	0	0	*θ*_2_
3(U)	*a*_3_	0	0	*θ*_3_
4(R)	0	90	*d*_4_	*θ*_4_
5(B)	0	−90	*d*_5_	*θ*_5_
6(T)	0	0	*d*_6_	*θ*_6_

The transformation matrix between two adjacent coordinate systems of the robot is *T*_*i*_. Positive kinematics can be obtained from Eq (1).

$$T_{1}T_{2}T_{3}T_{4}T_{5}T_{6}=T_{end}$$ (1)

Where $T_{end}=\left(\begin{array}{cccc} l_{x} & m_{x} & n_{x} & p_{x}\\ l_{y} & m_{y} & n_{y} & p_{y}\\ l_{z} & m_{z} & n_{z} & p_{z}\\ 0 & 0 & 0 & 0 \end{array}\right)$

By reversible transformation of Eq (1), $T_{3}T_{4}T_{5}={\left(T_{2}\right)}^{-1}{\left(T_{1}\right)}^{-1}T_{end}{\left(T_{6}\right)}^{-1}$ was obtained.

Right and left matrices, elements (3,4), (3,3), (3,2) were correspondingly equal, obtaining the following equations.

$$d_{6}\left(c_{1}n_{y}-n_{x}s_{1}\right)-c_{1}p_{y}+p_{x}s_{1}=d_{4}$$

$$n_{x}s_{1}-c_{1}n_{y}=c_{5}$$

$$-c_{6}\left(c_{1}m_{y}-m_{x}s_{1}\right)-s_{6}\left(c_{1}l_{y}-l_{x}s_{1}\right)=0$$

Where $s_{i}=sin\left(\theta_{i}\right),c_{i}=cos\left(\theta_{i}\right),s_{ij}=sin\left(\theta_{i}+\theta_{j}\right),c_{ij}=cos\left(\theta_{i}+\theta_{j}\right)$.

Then we can get Eqs (2)–(4).

$$x_{1}=\frac{-b\pm \sqrt{b^{2}-4ac}}{2a}$$

$$\theta_{1}=2tan^{-1}\left(x_{1}\right)$$ (2)

$$\theta_{5}=\pm cos^{-1}\left(n_{x}s_{1}-n_{y}c_{1}\right)$$ (3)

$$\theta_{6}=tan^{-1}\left(\frac{m_{x}s_{1}-c_{1}m_{y}}{c_{1}l_{y}-l_{x}s_{1}}\right)$$ (4)

Where $a=p_{y}-d_{6}n_{y}-d_{4}$, $b=2\left(p_{x}-d_{6}n_{x}\right)$, $c=d_{6}n_{y}-p_{y}-d_{4}$.

By reversible transformation of Eq (1), $T_{2}T_{3}T_{4}={\left(T_{1}\right)}^{-1}T_{end}{\left(T_{6}\right)}^{-1}{\left(T_{5}\right)}^{-1}$ was obtained.

Right and left matrices, elements (1,4), (2,4) were correspondingly equal, obtaining the following equations.

$$a_{2}c_{2}+a_{3}c_{23}=c_{1}p_{x}-d_{6}\left(c_{1}n_{x}+n_{y}s_{1}\right)-d_{5}\left(c_{6}\left(c_{1}m_{x}+m_{y}s_{1}\right)+s_{6}\left(c_{1}l_{x}+l_{y}s_{1}\right)\right)-a_{1}+p_{y}s_{1}$$

$$a_{2}s_{2}+a_{3}s_{23}=p_{z}-d_{6}n_{z}-d_{5}\left(c_{6}m_{z}+l_{z}s_{6}\right)$$

Then we can get Eqs (5)–(6).

$$\theta_{3}=\pm cos^{-1}\left(\frac{l^{2}+h^{2}-{a_{2}}^{2}-{a_{3}}^{2}}{2a_{2}a_{3}}\right)$$ (5)

$$x_{2}=\frac{m\pm \sqrt{m^{2}+n^{2}-l^{2}}}{l+n}$$

$$\theta_{2}=2\;tan^{-1}\left(x_{2}\right)$$ (6)

Where $l=c_{1}p_{x}-d_{6}\left(c_{1}n_{x}+n_{y}s_{1}\right)-d_{5}\left(c_{6}\left(c_{1}m_{x}+m_{y}s_{1}\right)+s_{6}\left(c_{1}l_{x}+l_{y}s_{1}\right)\right)-a_{1}+p_{y}s_{1}$, $h=p_{z}-d_{6}n_{z}-d_{5}\left(c_{6}m_{z}+l_{z}s_{6}\right)$, $m=-a_{3}s_{3}$, $n=a_{2}+a_{3}c_{3}$.

By reversible transformation of Eq (1), $T_{4}T_{5}={\left(T_{3}\right)}^{-1}{\left(T_{2}\right)}^{-1}{\left(T_{1}\right)}^{-1}T_{end}{\left(T_{6}\right)}^{-1}$ was obtained.

Right and left matrices, element (1,4) was correspondingly equal, obtaining the following equations.

$$d_{5}s_{4}=p_{z}s_{23}-d_{6}\left(n_{z}s_{23}+c_{1}n_{x}c_{23}+n_{y}s_{1}c_{23}\right)-a_{1}c_{23}-a_{3}-a_{2}c_{3}+c_{1}p_{x}c_{23}+p_{y}s_{1}c_{23}$$

Then we can get Eq (7).

$$\theta_{4}=\pm sin^{-1}\left(g\right)$$ (7)

Where

$$g=\frac{p_{z}s_{23}-d_{6}\left(n_{z}s_{23}+c_{1}n_{x}c_{23}+n_{y}s_{1}c_{23}\right)-a_{1}c_{23}-a_{3}-a_{2}c_{3}+c_{1}p_{x}c_{23}+p_{y}s_{1}c_{23}}{d_{5}}$$

### Inverse kinematics of oblique non-spherical wrist robot based on two optimization methods

**Fig 2:**
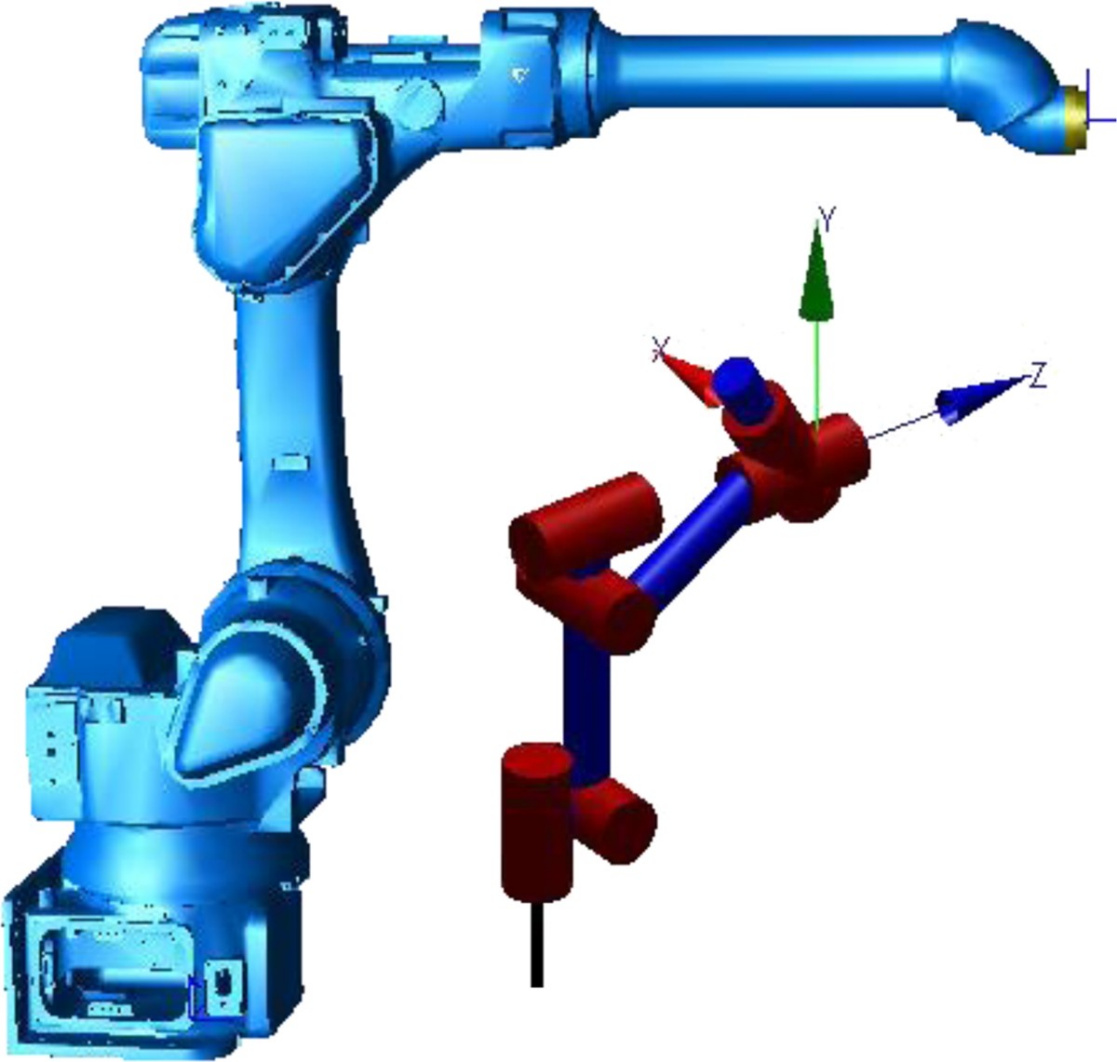
Model of oblique non-spherical wrist robot.

YASKAWA-EPX2800 (lemma wrist) was taken as an example in this paper. The model is shown in Fig 2, and DH parameters is shown in Table 2.

**Table 2:** DH parameters of oblique non-spherical wrist robot.

	$a_{i-1}\left(m\right)$	$\alpha_{i-1}{(}^{\circ })$	$d_{i}\left(m\right)$	$\theta_{i}{(}^{\circ })$
1(S)	*a*_1_	90	0	*θ*_1_
2(L)	*a*_2_	0	0	*θ*_2_
3(U)	*a*_3_	90	0	*θ*_3_
4(R)	0	*β*	*d*_4_	*θ*_4_
5(B)	0	−*β*	*d*_5_	*θ*_5_
6(T)	0	0	*d*_6_	*θ*_6_

#### Singularity analysis for oblique non-spherical wrist robot.

Differential transform method was used to construct the Jacobian matrix *J* of the robot in this structure. The robot in a singular configuration had less degree of freedom in operating space, and the determinant of *J* was 0. Therefore, the determinant could be used to determine the singular configuration. For simplified calculation, set the coordinate system $x_{5}-y_{5}-z_{5}$ to coincide with the coordinate system $x_{6}-y_{6}-z_{6}$. Via the complicated calculation and derivation, the determinant was obtained as shown in Eq (8).

$$\begin{array}{l} det\left(J\right)=a_{2}s\beta^{2}[(a_{1}a_{3}s_{3}-a_{1}c_{3}d_{4}+{a_{3}}^{2}c_{23}s_{3}-c_{3}{d_{4}}^{2}s_{23}+a_{3}d_{4}s_{3}s_{23}-a_{2}c_{2}c_{3}d_{4}-a_{3}c_{3}c_{23}d_{4}\\ a_{2}a_{3}c_{2}s_{3}-a_{3}c_{3}c_{23}c\beta d_{5}-c_{3}c\beta d_{4}d_{5}s_{23}+a_{1}d_{5}s_{3}s_{4}s\beta +a_{3}c_{2}{c_{4}}^{2}c\beta d_{5}-a_{1}c_{3}c\beta d_{5}{s_{4}}^{2}-\\ a_{2}c_{2}c_{3}c\beta d_{5}{s_{4}}^{2}+a_{2}c_{2}d_{5}s_{3}s_{4}s\beta +a_{3}c_{23}d_{5}s_{3}s_{4}s\beta +d_{4}d_{5}s_{3}s_{4}s_{23}s\beta )s_{5}+(a_{1}c_{3}+a_{3}c_{2}+\\ a_{2}c_{2}c_{3})c_{4}s_{4}d_{5}c_{5}] \end{array}$$ (8)

According to Eq (8), there were three cases when det(J)=0.

$$\{\begin{array}{c} s_{5}=0,s_{4}=0\\ s_{5}=0,c_{4}=0\\ s_{5}=0,c_{2}=0,c_{3}=0 \end{array}$$ (9)

By decomposing the Jacobian matrix *J*, *J* = *USV*^*T*^ was obtained, of which *S* was a diagonal matrix composed of non-zero singular values of *J*. The diagonal elements were arranged in descending order as $\sigma_{1}\geq \sigma_{2}\geq \sigma_{3}\geq \sigma_{4}\geq \sigma_{5}\geq \sigma_{6}$, of which the *σ*_6_ referred to the minimum singular value.

**Fig 3:**
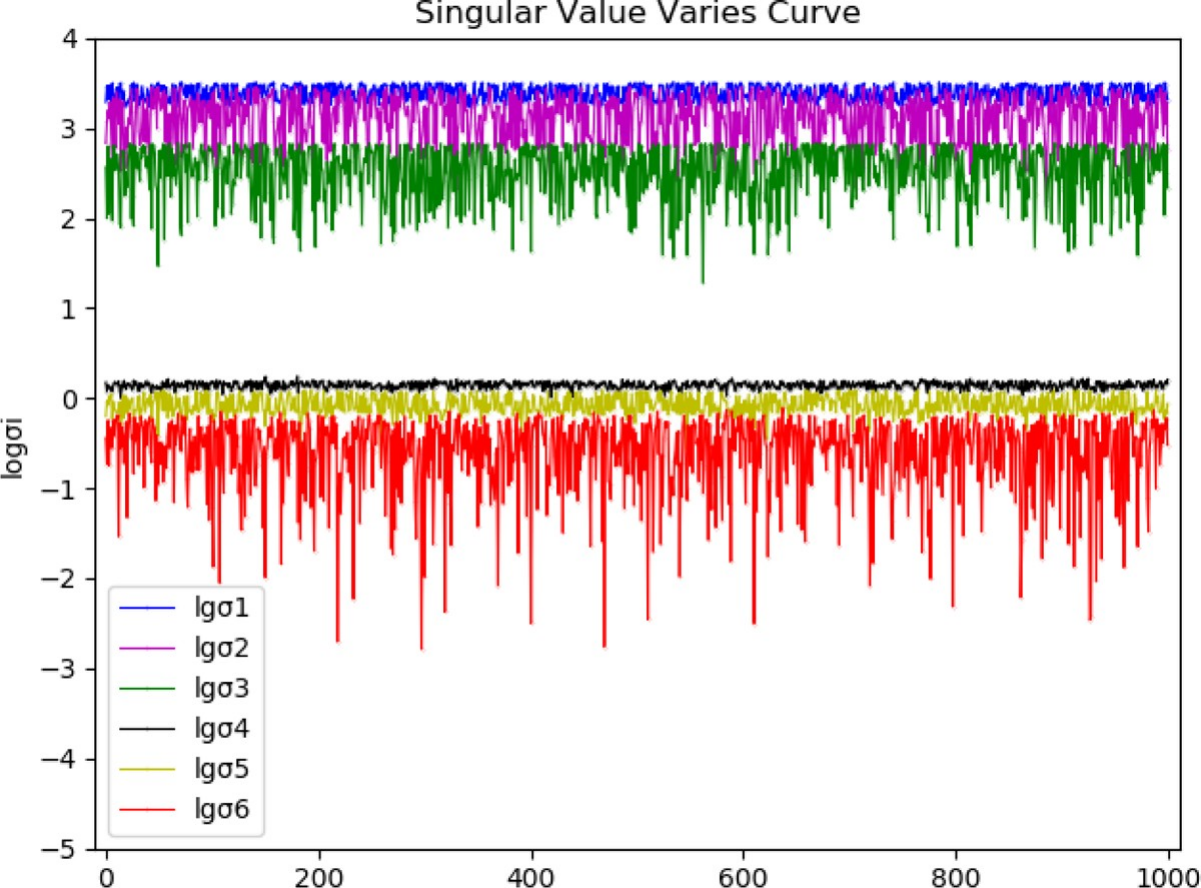
The characteristics of the singular values.

We then analyzed the relations between the singularities of the configuration and the singular value, and the characteristics of the singular values. One thousand positions were selected on a random basis to analyze the singular values, as shown in Fig 3. 10^6^ positions were selected at random. When the singular configuration and non-singular configuration, the mean singular value and variable coefficient were in statistics and shown in Table 3.

**Table 3:** Singular value comparison of singular points and non-singular points.

	Non-singular		Singular	
	mean	variation coefficient	mean	variation coefficient
*σ*_1_	2553.239	0.180	2524.623	0.183
*σ*_2_	1495.735	0.517	1464.438	0.517
*σ*_3_	399.138	0.505	386.052	0.538
*σ*_4_	1.394	0.086	1.389	0.091
*σ*_5_	0.861	0.224	0.975	0.239
*σ*_6_	0.347	0.566	1.639e^−14^	2.217

Fig 3 intuitively showed the bigger change in *σ*_2_, *σ*_3_, *σ*_6_, and smaller change in *σ*_1_, *σ*_4_, *σ*_5_, of which the *σ*_6_ had the greatest change. Therefore, in various damping iteration methods for analyzing the inverse kinematics, the method taking all singular values into account had a better effect than the method of determining damping factors only by the minimum singular value. The statistics data in Table 3 showed that the mean value of minimum singular values varied greatly from variable coefficients for singular configuration and non-singular configuration. Therefore, in analyzing the influence factors of the enhanced step length coefficient, the singularity of the configuration could be roughly represented by the minimum singular values.

#### Optimized elimination method.

The specific process of elimination method was as follows. By reversible transformation of Eq (1), it was obtained that:

$$T_{3}T_{4}T_{5}={\left(T_{2}\right)}^{-1}{\left(T_{1}\right)}^{-1}T_{end}{\left(T_{6}\right)}^{-1}$$

Right and left matrices, elements in column 3, 4 were correspondingly equal, obtaining Eq 6 unrelated to $\theta_{6}$.

$$p_{1}:c_{3}f_{1}+s_{3}f_{2}+a_{2}=c_{2}h_{1}+s_{2}h_{2}\;\left(e_{1}\right)$$

$$p_{2}:c_{3}f_{2}-s_{3}f_{1}=s_{2}h_{1}-c_{2}h_{2}\;\left(e_{2}\right)$$

$$p_{3}:f_{3}=h_{3}\;\left(e_{3}\right)$$

$$l_{1}:c_{3}r_{1}+s_{3}r_{2}=c_{2}n_{1}+s_{2}n_{2}\;\left(e_{4}\right)$$

$$l_{2}:s_{3}r_{1}-c_{3}r_{2}=c_{2}n_{2}-s_{2}n_{1}\;\left(e_{5}\right)$$

$$l_{3}:r_{3}=n_{3}\;\left(e_{6}\right)$$

Via the vector calculation of *p* and *l*, p⋅p, p⋅l, p×l, (p⋅p)l−2(p⋅l)p, Eq 8 were derived.

$${p_{1}}^{2}+{p_{2}}^{2}+{p_{3}}^{2}\;\left(e_{7}\right)p_{1}l_{1}+p_{2}l_{2}+p_{3}l_{3}\;\left(e_{8}\right)$$

$$p_{1}l_{2}-p_{2}l_{1}\;\left(e_{9}\right)p_{2}l_{3}-p_{3}l_{2}\;\left(e_{10}\right)p_{3}l_{1}-p_{1}l_{3}\;\left(e_{11}\right)$$

$$\left({p_{1}}^{2}+{p_{2}}^{2}+{p_{3}}^{2}\right)l_{1}-2\left(p_{1}l_{1}+p_{2}l_{2}+p_{3}l_{3}\right)p_{1}\;\left(e_{12}\right)$$

$$\left({p_{1}}^{2}+{p_{2}}^{2}+{p_{3}}^{2}\right)l_{2}-2\left(p_{1}l_{1}+p_{2}l_{2}+p_{3}l_{3}\right)p_{2}\;\left(e_{13}\right)$$

$$\left({p_{1}}^{2}+{p_{2}}^{2}+{p_{3}}^{2}\right)l_{3}-2\left(p_{1}l_{1}+p_{2}l_{2}+p_{3}l_{3}\right)p_{3}\;\left(e_{14}\right)$$

Via eliminating the Eq 14 $e_{1}\sim e_{14}$ by *θ*_1_, *θ*_2_, we obtained Eq 6. Wrote Eq 6 in the form of a matrix equation, obtaining the matrix equation

$$\left(\Sigma \right)v_{1}=0$$ (10)

Of which $v_{1}={\left[s_{4}s_{5}s_{4}c_{5}c_{4}s_{5}c_{4}c_{5}s_{4}c_{4}s_{5}c_{5}\;1\right]}^{T}$, (*Σ*) referred to a 6*9 matrix containing only unknowns *s*_3_, *c*_3_. Converted *s*_3_, *c*_3_, *s*_4_, *c*_4_, *s*_5_, *c*_5_ into $s_{3}=\frac{2x_{3}}{1+{x_{3}}^{2}}$, $c_{3}=\frac{1-{x_{3}}^{2}}{1+{x_{3}}^{2}}$, $s_{4}=\frac{2x_{4}}{1+{x_{4}}^{2}}$, $c_{4}=\frac{1-{x_{4}}^{2}}{1+{x_{4}}^{2}}$, $s_{5}=\frac{2x_{5}}{1+{x_{5}}^{2}}$, $c_{5}=\frac{1-{x_{5}}^{2}}{1+{x_{5}}^{2}}$, and obtained the matrix equation

$$\left(\Sigma^{\prime }\right)v_{2}=0$$ (11)

Of which $v_{2}={\left[{x_{4}}^{2}{x_{5}}^{2}{x_{4}}^{2}x_{5}{x_{4}}^{2}x_{4}{x_{5}}^{2}x_{4}x_{5}x_{4}{x_{5}}^{2}x_{5}1\right]}^{T}$, (*Σ*′) referred to a 6*9 matrix containing only unknowns *x*_3_. Multiplied each line of the matrix equation by *x*_4_ and merged the equation obtained with the original equation, to get the matrix equation

$$\left(\Sigma^{\prime \prime }\right)v=0$$ (12)

Of which $v={\left[{x_{4}}^{3}{x_{5}}^{2}{x_{4}}^{3}x_{5}{x_{4}}^{3}{x_{4}}^{2}{x_{5}}^{2}{x_{4}}^{2}x_{5}{x_{4}}^{2}x_{4}{x_{5}}^{2}x_{4}x_{5}x_{4}{x_{5}}^{2}x_{5}\;1\right]}^{T}$, (*Σ*′′) referred to a 12*12 matrix with only unknowns *x*_3_. This was an over-constrained linear system. The coefficient matrix (*Σ*′′) should be singular for the equation to have a solution, and the determinant of the coefficient matrix was a 16th degree polynomial in one variable regarding *x*_3_. The roots of the polynomial were corresponded to the solution to inverse kinematics. For improving the solving speed, the problem of solving 16th degree polynomial was simplified to find the matrix eigenvalues and eigenvectors. (*Σ*′′) can be written as $A{x_{3}}^{2}+Bx_{3}+C$, and the Eq (12) can be written as

$$\left(A{x_{3}}^{2}+Bx_{3}+C\right)v=0$$ (13)

*A*, *B*, *C* were a known 12*12 matrix, the Eq (13) can be written as

$$\left[\begin{array}{cc} 0 & I\\ -A^{-1}C & -A^{-1}B \end{array}\right]\left[\begin{array}{c} v\\ x_{3}v \end{array}\right]=x_{3}\left[\begin{array}{c} v\\ x_{3}v \end{array}\right]\;\text{i}.\text{e}.\;M\left[\begin{array}{c} v\\ x_{3}v \end{array}\right]=x_{3}\left[\begin{array}{c} v\\ x_{3}v \end{array}\right]$$ (14)

The matrix eigenvalues of *M* were *x*_3_, and the eigenvectors were $V=\left[\begin{array}{c} v\\ x_{3}v \end{array}\right]/\left\|\left[\begin{array}{c} v\\ x_{3}v \end{array}\right]\right\|$. *θ*_3_ can be obtained from $\theta_{3}=2tan^{-1}\left(x_{3}\right)$.

In this paper, the elimination method is optimized and supplemented in three points.

1. The paper presented the specific process of Eq 6 obtained by eliminating Eq 14 by $e_{1}\sim e_{14}$. First, the expression *s*_1_, *c*_1_ obtained from two equations *e*_3_, *e*_6_ was substituted into *e*_7_, *e*_8_, *e*_9_, *e*_14_, getting 4 equations. Two equations would be obtained by simplifying the *e*_1_, *e*_2_, *e*_4_, *e*_5_, *e*_10_, *e*_11_, *e*_12_, *e*_13_ via Eqs (15)–(16). There were no specific simplified formulas in other literatures.
  $$\frac{{\mu_{1}}^{2}}{2a_{1}}\left(e_{12}-\delta_{1}e_{4}+\delta_{2}e_{1}\right)-\lambda_{1}\mu_{1}e_{10}+\mu_{1}we_{2}-\mu_{1}\left(r-d_{1}\right)e_{5}=0$$ (15)
  $$\frac{{\mu_{1}}^{2}}{2a_{1}}\left(e_{13}-\delta_{1}e_{5}+\delta_{2}e_{2}\right)-\lambda_{1}\mu_{1}e_{11}-\mu_{1}we_{1}+\mu_{1}\left(r-d_{1}\right)e_{4}=0$$ (16)
  Where $\lambda_{i}=cos\alpha_{i}$, $\mu_{i}=sin\alpha_{i}$, $\delta_{1}=p^{2}+q^{2}+{\left(r-d_{1}\right)}^{2}-{a_{1}}^{2}$, $\delta_{2}=2\left(pu+qv+\left(r-d_{1}\right)w\right)$
2. In other documents, the final expression of the matrix *A*, *B*, *C* was not deduced, and the solution required to be made in several steps. For improving the solving speed, the paper adopted complex coefficient extraction and simplification to derive the final expression of matrix *A*, *B*, *C*. Maximized the simplification of the whole solution process for achieving one-step solution of the matrix *M*. This was also the greatest contribution of the paper to eliminate optimization. The expression of *A*, *B*, *C* is shown in the appendix and refer to the uploaded matlab code. Refer to S1 Table. https://github.com/Wangxiaoqi1031/robot
3. In this paper, the solution formula in the simplest form of other joint angles *θ*_1_, *θ*_2_, *θ*_4_, *θ*_5_, *θ*_6_ was introduced to integrate the whole method. Through a lot of experiments, taking the item with a large absolute value in the *M* matrix eigenvector as ratio, to get more accurate *x*_4_, *x*_5_. Via analysis, the maximum value may be *v*_1_, *v*_3_, *v*_10_, *v*_12_, and the formula *θ*_4_, *θ*_5_ obtained was shown as Eq (17).
  $$\{\begin{array}{ccc} \theta_{4}=tan^{-1}\left(\frac{v_{1}}{v_{4}}\right) & \theta_{5}=tan^{-1}\left(\frac{v_{1}}{v_{2}}\right) & max\left(v_{i}\right)=\left|v_{1}\right|\\ \theta_{4}=tan^{-1}\left(\frac{v_{3}}{v_{6}}\right) & \theta_{5}=tan^{-1}\left(\frac{v_{2}}{v_{3}}\right) & max\left(v_{i}\right)=\left|v_{3}\right|\\ \theta_{4}=tan^{-1}\left(\frac{v_{7}}{v_{10}}\right) & \theta_{5}=tan^{-1}\left(\frac{v_{10}}{v_{11}}\right) & max\left(v_{i}\right)=\left|v_{10}\right|\\ \theta_{4}=tan^{-1}\left(\frac{v_{9}}{v_{12}}\right) & \theta_{5}=tan^{-1}\left(\frac{v_{11}}{v_{12}}\right) & max\left(v_{i}\right)=\left|v_{12}\right| \end{array}$$ (17)
  $$s_{1}=\frac{\left(p_{y}-d_{6}n_{y}\right)\left(c_{4}c\beta s\beta +s_{4}s_{5}s\beta -c_{4}c_{5}c\beta s\beta \right)-c_{4}d_{5}n_{y}s\beta }{n_{y}p_{x}-n_{x}p_{y}}$$
  $$s_{2}=\frac{h_{1}e_{2}+h_{2}e_{1}}{{h_{1}}^{2}+{h_{2}}^{2}}$$
  $$\theta_{1}=sin^{-1}\left(s_{1}\right),\;\theta_{2}=sin^{-1}\left(s_{2}\right)$$ (18)
  $$x_{6}=\frac{m\pm \sqrt{m^{2}+n^{2}-l^{2}}}{l+n}$$
  $$\theta_{6}=2tan^{-1}\left(x_{6}\right)$$ (19)

Where $l=\frac{d_{5}c\beta \left(n_{y}c_{1}-n_{x}s_{1}\right)+d_{6}\left(n_{y}c_{1}-n_{x}s_{1}\right)-p_{y}c_{1}+p_{x}s_{1}}{d_{5}s\beta }$, $n=m_{y}c_{1}-m_{x}s_{1}$, $m=l_{y}c_{1}-l_{x}s_{1}$

#### Optimized Gaussian damped least square method.

The Jacobian matrix pseudo-inverse method proposed by Whitney [3] is most widely used for solving robot inverse kinematics. The joint velocity of the robot is obtained from the speed of the end effector.

$$\dot{\theta }=J^{+}\dot{X}$$ (20)

Where *J*^+^ is the pseudo inverse of the robot Jacobian matrix *J*. This method gives the best possible solutions that minimize joint velocity norm ${\left\|\dot{\theta }\right\|}^{2}$ and the end-effector tracking error ${\left\|\dot{X}-J\dot{\theta }\right\|}^{.2}$. However, the method is unstable near singularities. *SVD* decomposing the Jacobian matrix to get

$$J=USV^{T}$$ (21)

*S* is a diagonal matrix formed by the singular values of *J*.which are arranged in descending order, $\sigma_{1}\geq \sigma_{2}\geq \cdots \sigma_{6}$. Hence, the joint speed could be computed as follows.

$$\dot{\theta }=J^{+}\dot{X}=\Sigma_{i=1}^{6}\frac{1}{\sigma_{i}}VU^{T}\dot{X}$$ (22)

While a robot approaching a singular configuration, the smallest singular value reaches 0 and it causes infinite joint velocities as implied in (22). Therefore, damped least squares (DLS) introduces a damping factor, balances the precision of the solution and the increase in joint velocities near singularities by minimizing ${\left\|J\dot{\theta }.-\dot{X}\right\|}^{.2}+\lambda^{2}{\left\|{\dot{\theta }}^{*}\right\|}^{2}$. The joint speed could be computed as follows.

$$\dot{\theta }*=\Sigma_{i=1}^{6}\frac{\sigma_{i}}{{\sigma_{i}}^{2}+\lambda^{2}}VU^{T}\dot{X}$$ (23)

Multiple methods are generated from damping least square method and selecting strategies based on different damping factors. A constant damping factor was proposed in DLS method. The relative relation between the end-effecter position and the target position was considered in SDLS method, and a piecewise function was adopted to determine the damping factor. Gauss damping least square (GDLS) method refers to determine the damping factors with the following functions, based on the Gaussian distribution of the damping factor.

$$\lambda_{i}=\lambda_{max}\cdot e^{-{\left(\sigma_{i}/\varepsilon \right)}^{2}}$$ (24)

**Fig 4:**
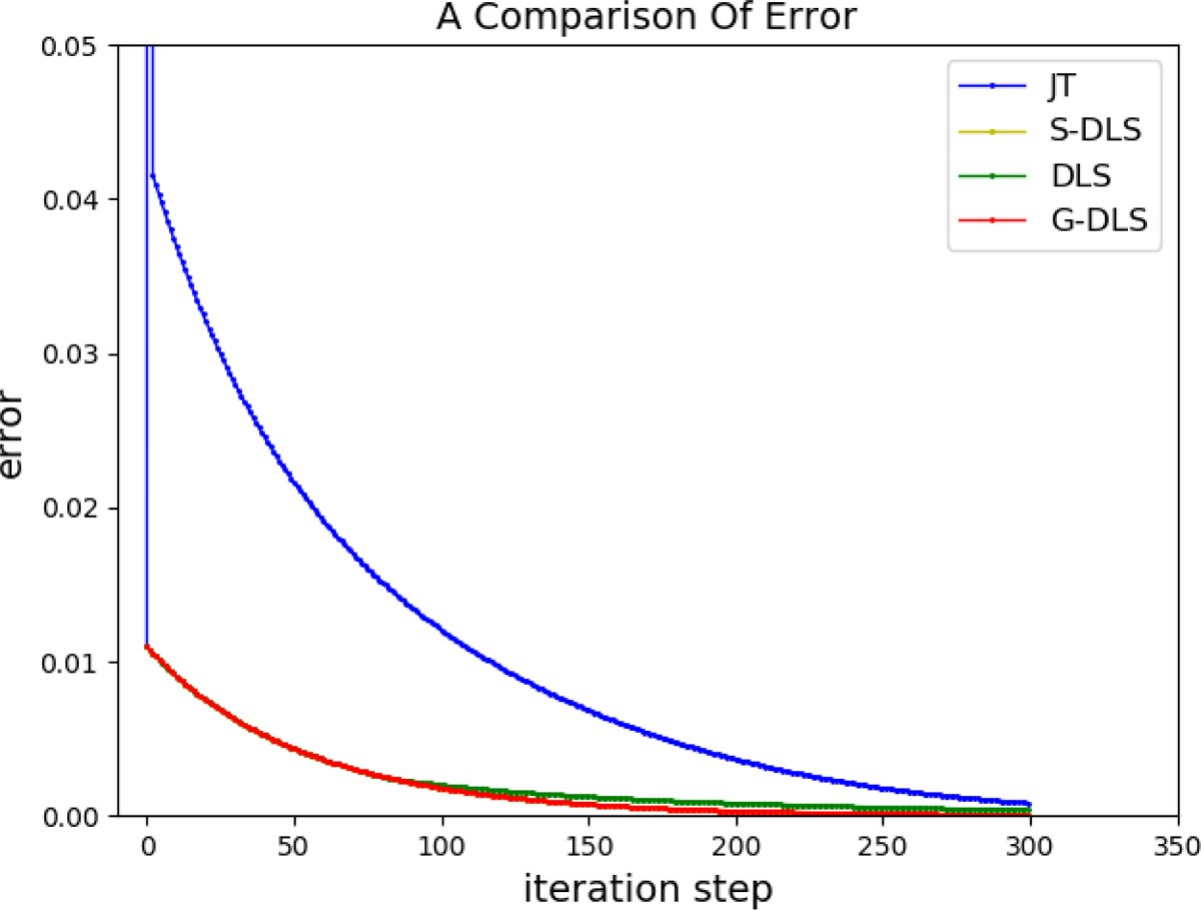
Comparison of four methods.

Where *λ*_*max*_ is the maximum value of the damping factor and *ε* is a scalar quantity indicating region of singularities and *σ*_*i*_ is singular value. GDLS is more effective than other methods that select constant and piecewise function damping factors. On one hand, each singular value corresponds to a damping factor, so that damping only act on singular vectors, to prevent the introduction of unnecessary damping and reduce the errors. On the other hand, when the robot is in close proximity to singular configuration, the method allows to make the joint velocity continuously change from undamped to damped, for more stable motion. The *λ*_*max*_, *ε* of the robot configuration in this paper was determined based on the genetic algorithm in the literature [8] and obtained *λ*_*max*_ = 0.071, *ε* = 0.051. The Jacobian matrix pseudo-inverse method *J*^*T*^, DLS,SDLS and GDLS were used to analyze the inverse kinematics when close to the singular configuration and compare the performance of the four methods, as shown in Fig 4.

Fig 4 showed that when the robot approaches a singular configuration, compared with the Jacobian matrix pseudo-inverse method, the damped least square method prevented oscillation and effectively controlled the joint velocity, making the more stable motion. SDLS had similar effects as GDLS had, which reduced unnecessary damping and errors, compared with DLS.

**Fig 5:**
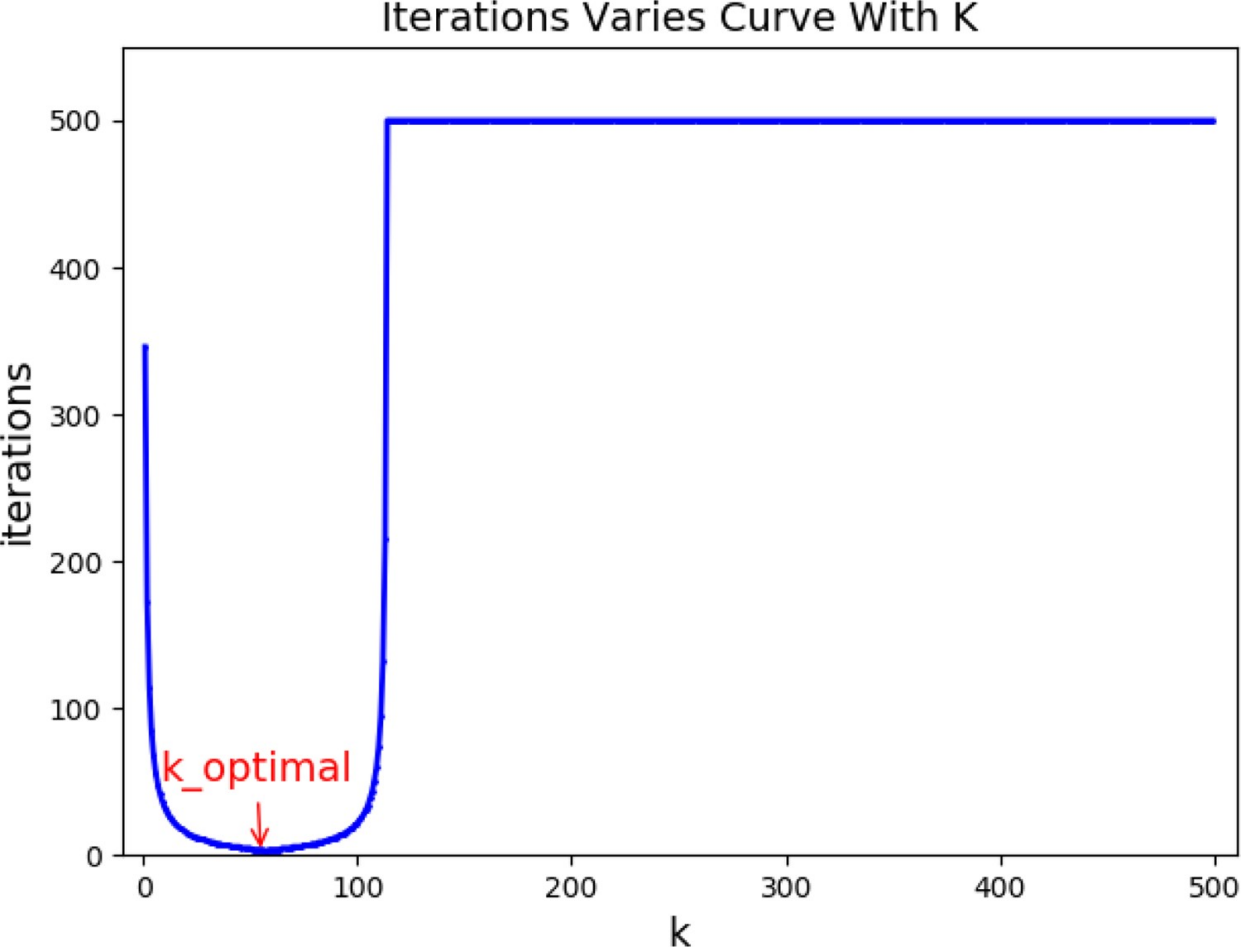
Number of iterations varies with k.

DLS series methods including GDLS can be used to solve the IK problem of robot, and can ensure the stability of motion by controlling the joint speed. However, hundreds of iterations are needed to obtain the solution meeting the accuracy requirement in the solving process, which cannot achieve the goal of high-speed to solve IK. In the iteration process, it is found that the speed of approaching the iteration convergence can be improved through multiplying the iterative increment *dθ* by a coefficient *k*, achieving faster iteration convergence. The change of the number of iterations with *k* can be usually obtained as shown in Fig 5 in the process of solving with GDLS method. Therefore, we introduce the concepts of enhanced step length coefficient and optimal enhanced step length coefficient.

Definition: During the iteration, change *θ*_*cur*_ = *θ*_*cur*_ + *dθ* into *θ*_*cur*_ = *θ*_*cur*_ + *kdθ*, to make the iteration converge faster, and *k* is called the enhanced step length coefficient. The number of iterations reached the least, when *k* was assigned a value, and the value was defined as the optimal enhanced step length coefficient *k*_*optimal*.

**Fig 6:**
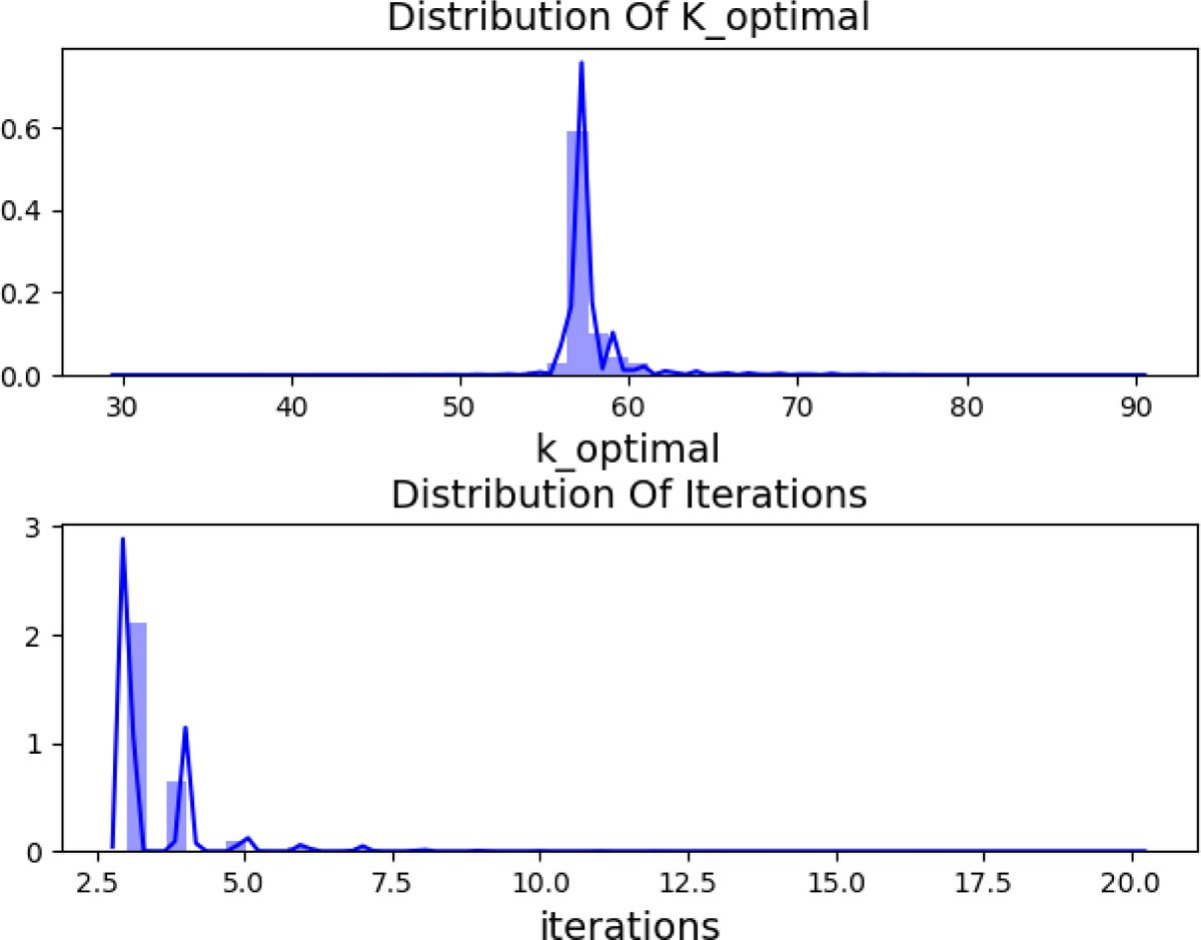
Distribution of *k_optimal* and iterations number.

To study the distribution of *k*_*optimal* and the range of the minimum number of iterations. We selected randomly 10^5^ points and controlled the initial and target position within a certain range. The probability distribution of *k*_*optimal* and the minimum number of iterations was obtained and shown in Fig 6. Distribution statistics data of *k*_*optimal* were shown in Table 4.

**Table 4:** Probability of *k_optimal* in different intervals.

k_optimal	Probability(%)
[30,55)	0.602
[55,60]	94.614
(60,65)	2.782
[65,90]	2.002

Fig 6 and Table 4 showed that the *k*_*optimal* was mainly distributed between [30,90] and centrally distributed between [55,60). If proper *k* was selected, the number of iterations can be controlled within 20 times.

**Fig 7:**
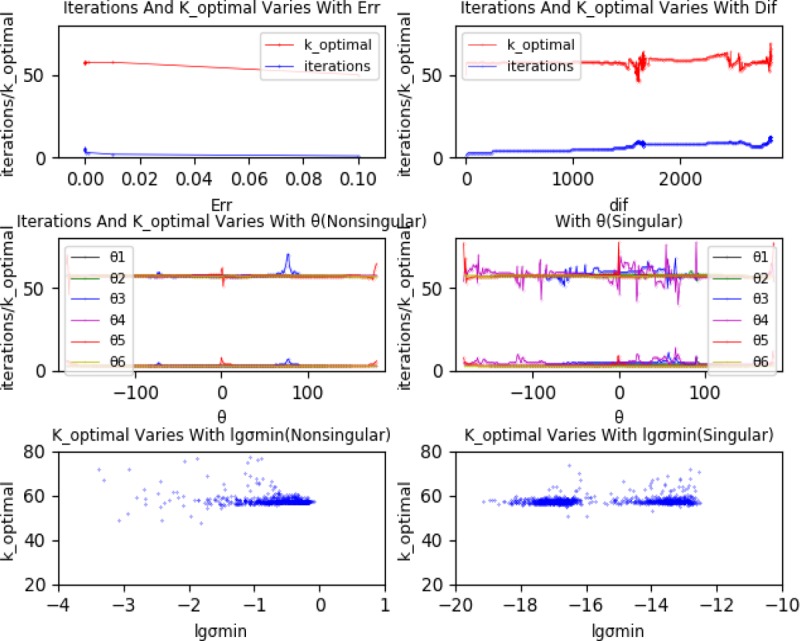
Influencing factors of *k_optimal*.

To study the influencing factors of *k*_*optimal*, the iterative process was analyzed and the supposed possible factors to be: The difference value *dif* between current position *T*_*cur*_ and terminal position *T*_*end*_, iterative convergence error *E*_*rr*_, initial iteration value *θ*_*i*_ and singularity. Via the analysis on the singularity in section 3.1, the minimum singular value *σ*_*m*_ was adopted to represent the singularity. When other conditions remained unchanged, we got and the variations of *k*_*optimal* and the minimum number of iterations with *dif*, *E*_*rr*_, *θ*_*i*_, *σ*_*m*_ were shown in Fig 7.

From Fig 7, when *E*_*rr*_ ≤ 0.01, there was almost no influences of *E*_*rr*_ on *k*_*optimal*. When *dif* changed over a larger scale, it did affect *k*_*optimal*. *θ*_3_ and *θ*_5_ had greater influence on *k*_*optimal* when the robot was far away from the singular configuration. When the robot approached the singular configuration, *θ*_2_, *θ*_3_, *θ*_4_ and *θ*_5_ had greater influence on *k*_*optimal*. After repeated experiments, we get that not every group of data could produce the same change rule and curve, while this section was intended to determine the influence factors of *k*_*optimal*, so the curve shape can be neglected. The singularity had no direct effect on *k*_*optimal*. Therefore, conclusion comes that the initial iteration value *θ*_*i*_ has a great impact on *k*_*optimal*.

Based on the analysis on the influencing factors of *k*_*optimal*. The initial iteration value *θ*_*i*_ was used as input and *k*_*optimal* as the output, and the *k*_*optimal* of each iteration was determined by the classification and regression models of machine learning. We selected 10^5^ random positions as research data and changed the initial iteration value *θ*_*i*_ to get *k*_*optimal* when other conditions remained unchanged.

- Classification method

According to the distribution of *k*_*optimal* in Table VI, we divided the values of k_optimal into four classes [30,55), [55,60], (60,65), [65,90]. It can be seen from Fig 5 that the number of iterations changes with *k* continuously and gradually decreases when *k* approaches *k*_*optimal*. Therefore, a slight difference between the final selected *k* value and *k*_*optimal* is acceptable, and the number of iterations will be close to the minimum. The initial iteration value *θ*_*i*_ was used as input and the classification of *k*_*optimal* used as the output, and the classification models of machine learning were used for classifying. The performance was evaluated by OA (overall accuracy), ECA (each class accuracy), AA (average accuracy) and TT (training time). Seven models with better performance were selected from various models for comparison, as in Table 5.

**Table 5:** Performance comparison of classification models.

Model	OA	ECA	AA	TT
KNN	0.89	[0.73 0.91 0.38 0.70]	0.68	0.115
NB	0.88	[0.69 0.89 0.00 0.73]	0.58	**0.016**
LR	0.87	[0.82 0.87 0.00 0.75]	0.61	0.182
RF	0.97	[0.95 0.97 0.83 0.93]	0.92	0.069
DT	**0.99**	[0.98 **0.99 0.95 0.97**]	**0.97**	0.021
GBDT	0.97	[**1.00** 0.98 0.88 0.94]	0.95	4.792
SVM	0.85	[0.00 0.85 0.00 0.00]	0.21	69.87

Table 5 showed that the overall and the average accuracy of the DT model were the highest. The ECA obtained from DT and GBDT were relatively high. NB model featured the shortest training time. On the whole, DT model had the best classification result. After classification, in the iteration process, the median of each range is taken for the value of *k*_*optimal* of each class, which is *k*_*optimal* = 42.5,57.5,62.5,77.5.

- Regression method

The initial iteration value *θ*_*i*_ was used as input and the *k*_*optimal* was used as the output, and the regression models of machine learning were used for analysis. The paper used three commonly used statistical indicators including root mean square error (RMSE), coefficient of determination (*R*^2^) and mean absolute error (MAE) to evaluate the performance of the model.

$$RMSE=\sqrt{\frac{1}{n}\Sigma_{i=1}^{n}{\left(Y_{i,m}-Y_{i,e}\right)}^{2}}$$ (25)

$$R^{2}=\frac{\Sigma_{i=1}^{n}{\left(Y_{i,m}-Y_{i,e}\right)}^{2}}{\Sigma_{i=1}^{n}{\left(Y_{i,m}-{\bar{Y}}_{i,m}\right)}^{2}}$$ (26)

$$MAE=\frac{1}{n}\Sigma_{i=1}^{n}\left|Y_{i,m}-Y_{i,e}\right|$$ (27)

**Fig 8:**
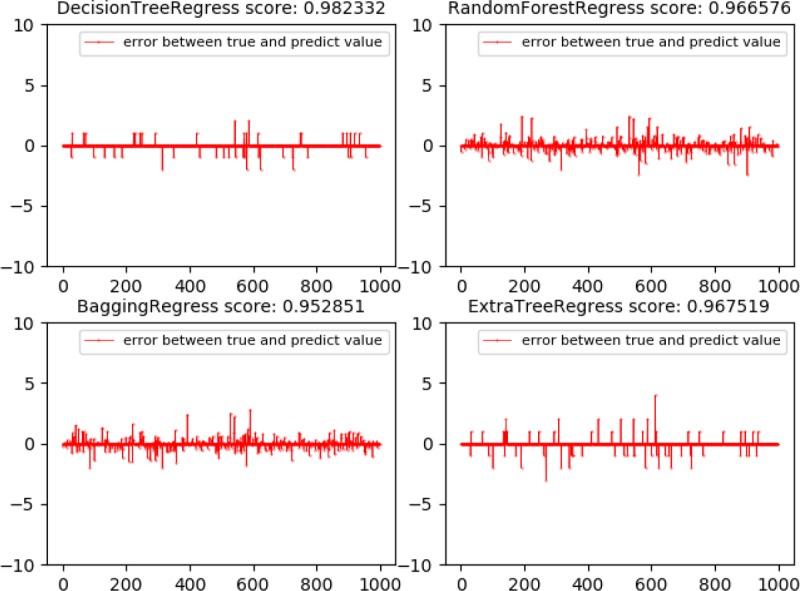
Regression fitting error curve of four models.

Three indicators were calculated according to Eqs (25)–(27). Five models with better performance were selected from various models for comparison, as shown in Table 6, of which four with better results were selected and 1000 groups of data were taken at random to generate the regression fitting error curve, as shown in Fig 8.

**Table 6:** Performance comparison of regression models.

Model	Training			Testing		
	*R*^2^	*RMSE*	*MAE*	*R*^2^	*RMSE*	*MAE*
DT	**1.000**	**0.000**	**0.000**	**0.982**	**0.340**	**0.064**
RF	0.988	0.253	0.072	0.945	0.599	0.198
Bagging	0.988	0.256	0.071	0.949	0.576	0.193
ETR	0.999	0.038	**0.000**	0.933	0.656	0.101
XBGboost	0.702	1.282	0.456	0.561	1.690	0.572

Table 6 showed that the DT model featured the best results for both training and testing samples. It could also be seen intuitively from Fig 8 that DT model had the best fitting effect. The DT model adopted cost complexity pruning (CCP) method and adjusted relevant parameters to avoid overfitting. The flow chart of the optimized Gauss damped least squares method are as follows.

**Fig 9:**
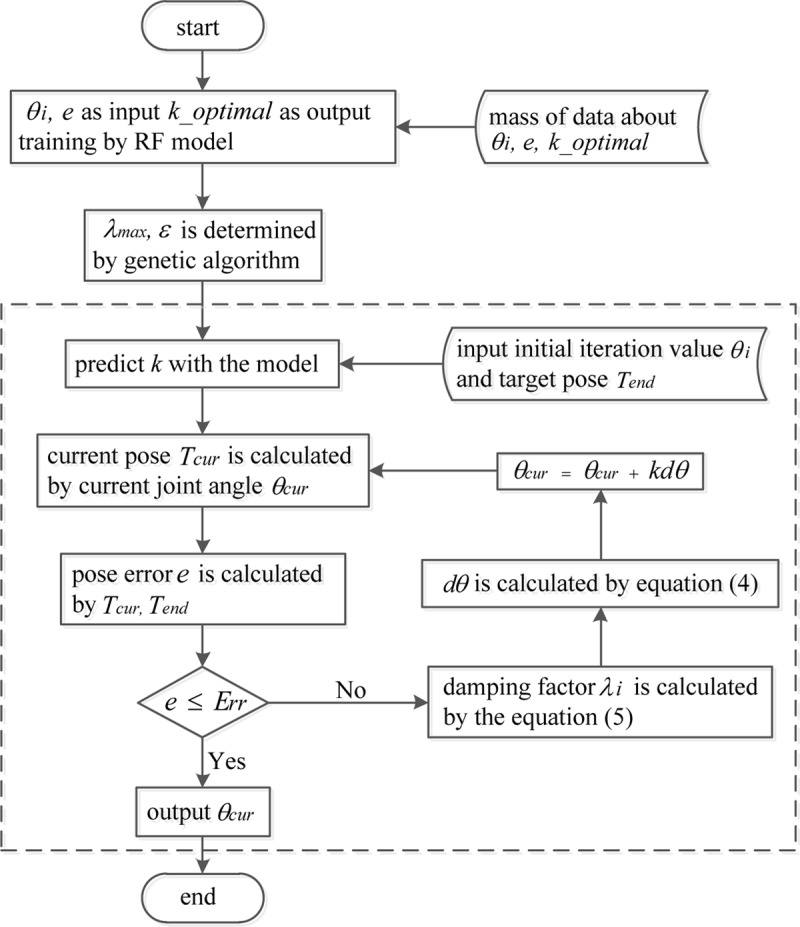
The flowchart of the optimized method.

The flow chart of the optimized algorithm is shown in Fig 9.

## Results and discussion

### Examples of orthogonal non-spherical wrist robot

The D-H parameters are *a*_1_ = 0.3, *a*_2_ = 0.85, *a*_3_ = 0.844, *d*_4_ = 0.165, *d*_5_ = 0.141, *d*_6_ = 0.093. We set *θ*_1_ = 30°, *θ*_2_ = 70°, *θ*_3_ = 60°, *θ*_4_ = 140°, *θ*_5_ = −40°, *θ*_6_ = 40°. Then we can get $T_{end}={\left[T_{1},T_{2},T_{3},T_{4}\right]}^{T}$. Where $T_{1}=\left[-0.80287234,-0.45682599,0.38302222,14.723871\right];$

$$T_{2}=\left[0.10504046,-0.74084306,-0.66341395,-264.28809\right];$$

$$T_{3}=\left[-0.586824089,0.492403877,-0.642787610,1416.60239\right];T_{4}=\left[0,0,0,1\right]$$

According to the Eqs (2)–(7), we can get *θ*_1_ ~ *θ*_6_. All the results that meet the accuracy requirements are as shown in Table 7.

**Table 7:** Results that meet the accuracy requirements.

	*θ*_1_(°)	*θ*_2_(°)	*θ*_3_(°)	*θ*_4_(°)	*θ*_5_(°)	*θ*_6_(°)	err
1	-41.778451	64.145049	72.666641	1.7309941	76.140837	-61.947867	1.4993e^−12^
2	-41.778451	138.49267	-72.666641	72.716655	76.140837	-61.947867	1.8000e^−12^
**3**	**30.000000**	**69.999999**	**60.000000**	**139.99999**	**-40.000000**	**39.999999**	**3.8979e**^**−13**^
4	30.000000	131.31962	-60.000000	198.68037	-40.000000	39.999999	5.9746e^−13^

For similar configurations, the formulas are universal.

### Examples of oblique non-spherical wrist robot

#### Examples and properties of optimized elimination method.

The D-H parameters of the configuration were *a*_1_ = 0.25, *a*_2_ = 0.95, *a*_3_ = 0.3, *d*_4_ = 1.55, *d*_5_ = 0.114, *d*_6_ = 0.123, *β* = 60°, we set *θ*_1_ = 14°, *θ*_2_ = 29.7°, *θ*_3_ = −45°, *θ*_4_ = 71°, *θ*_5_ = −63°, *θ*_6_ = 100°, and let the terminal position matrix be $T_{end}={\left[T_{1},T_{2},T_{3},T_{4}\right]}^{T}$.

Where $T_{1}=\left[-0.53060777-0.71765135,0.4510343,1.047652\right];$

$$T_{2}=\left[-0.79429474,0.23523209,-0.56014439,0.21160551\right];$$

$$T_{3}=\left[0.29589063,-0.65547114,-0.69484266,-1.2686177\right];T_{4}=\left[0,0,0,1\right]$$

The matrix *A*, *B*,*C* can be directly obtained from the formula in the appendix. The matrix *M* was obtained from the formula (14) and the eigenvalues and eigenvectors of the matrix were obtained. Real eigenvalue of *M* matrix was [-61.880055, 39.835519, -6.2262997, -5.0896338, -0.41421356, -0.34282825, -0.21892882, -0.17065825]. Then other joint variables were obtained from the equation (17)–(19). All solutions that meet the accuracy requirements, as shown in Table 8.

**Table 8:** Results that meet the accuracy requirements.

	*θ*_1_	*θ*_2_	*θ*_3_	*θ*_4_	*θ*_5_	*θ*_6_	err
1	-162.89741	156.18595	-178.14833	-76.120407	-48.798102	47.154357	3.03917e^−14^
2	-163.75277	154.00112	177.12399	85.181957	49.016271	-168.81112	2.64275e^−14^
3	20.011962	-142.53364	-161.7514	140.31013	-63.624205	8.6364123	4.26276e^−14^
4	14.018839	-140.47799	-157.76849	-71.20786	62.629725	144.74019	2.01718e^−14^
**5**	**14.000000**	**29.7000000**	**-45.000000**	**71.000000**	**-63.00000**	**100.000000**	**2.51878e**^**−11**^
6	19.565127	27.505394	-37.846326	-133.49287	58.583668	-117.21089	1.86615e^−11^
7	-166.21313	-68.60891	-24.697729	111.37032	88.933046	109.22533	5.38294e^−11^
8	-158.97354	-69.890768	-19.369394	-17.484555	-91.606294	-9.1357937	7.86953e^−11^

For analyzing the algorithm performance, we selected 10^6^ positions of the robot in the reachable workspace at random, to find a solution by the above optimized elimination method. And the testing environment was (Intel(R) Xeon CPU2.8GHz, python). The statistical results showed that the average solving speed was reduced from 11-13ms before optimization to 1.48ms, and the solving speed was reduced to 20% of the original, the average accuracy of solution reached 2.3205e^−13^. During solving by elimination, the proportion of the points that cannot be solved accurately since the *A* matrix was singular or the robot approached the singular configuration was 0.086%, which can be solved by the following solutions.

1. In the case of A is singular, finding inverse is changed to find pseudo-inverse.
2. According to the singularity analysis in Section 3.1, there is no singular regions in trajectory planning.
3. Points that cannot be solved accurately can be processed by iteration method.

#### Examples and properties of optimized Gaussian damped least square method.

For comparing the solving performance of the classification and regression, we selected 10^5^ positions at random and adopted DT model to predict the *k* value separately, and solved it in accordance with the above steps. The resulting statistics, as shown in Table 9.

**Table 9:** Performance analysis of two methods of machine learning.

method	Average number of iterations	Average forecast time(s)	Iteration more than 10	Iteration more than 20
classification	6.55	7.962e-07	1.68%	0.08%
regression	3.6414	9.8465e-07	0.29%	0.05%

Table 9 gave that the regression method featured less average number of iterations. The two methods had almost the same average prediction time, and the average prediction time was negligible compared with the solution time. The regression method had a lower proportion of iterations over 10 times and over 20 times, less than 0.1%. On the whole, the regression method had better effects than classification.

For comparing the solving performance of the methods before and after optimization, the Gauss damped least square method and the optimized method in the paper were used for solving separately. A complete experimental process was: we randomly selected 10^5^ data points as the original data set, each data point contains the initial iteration value and *k*_*optimal*. The collected data set was used to train a DecisionTree model through supervised learning to obtain a model that can be used to predict *k*_*optimal*. Then 10^4^ data points were randomly selected in the robot workspace, that is, the initial iteration values were randomly selected, and the IK was solved by the GDLS method before and after optimization. Then we counted the number of iterations and the solution time. The above experiment was repeated 10 times and the statistics were obtained. The best, worst, and average cases in the statistical repeat experiments were shown in Table 10.

**Table 10:** Performance analysis before and after optimization.

method	Number of iterations			Solution time (ms)		
	Maximum	Minimum	Average	Maximum	Minimum	Average
GDLS	137.1	119.8	125.3	3.813	3.309	3.491
Optimized GDLS	4.143	2.918	3.641	0.1467	0.1132	0.1298

Table 10 showed that the average iteration times of the optimized method was obviously reduced, and the average solution time was reduced to 5% of the original, the average solution time of optimized method was close to the solution time of the analytic solution of the general configuration. The optimized method in this paper was very effective for improving the solving speed.

**Fig 10:**
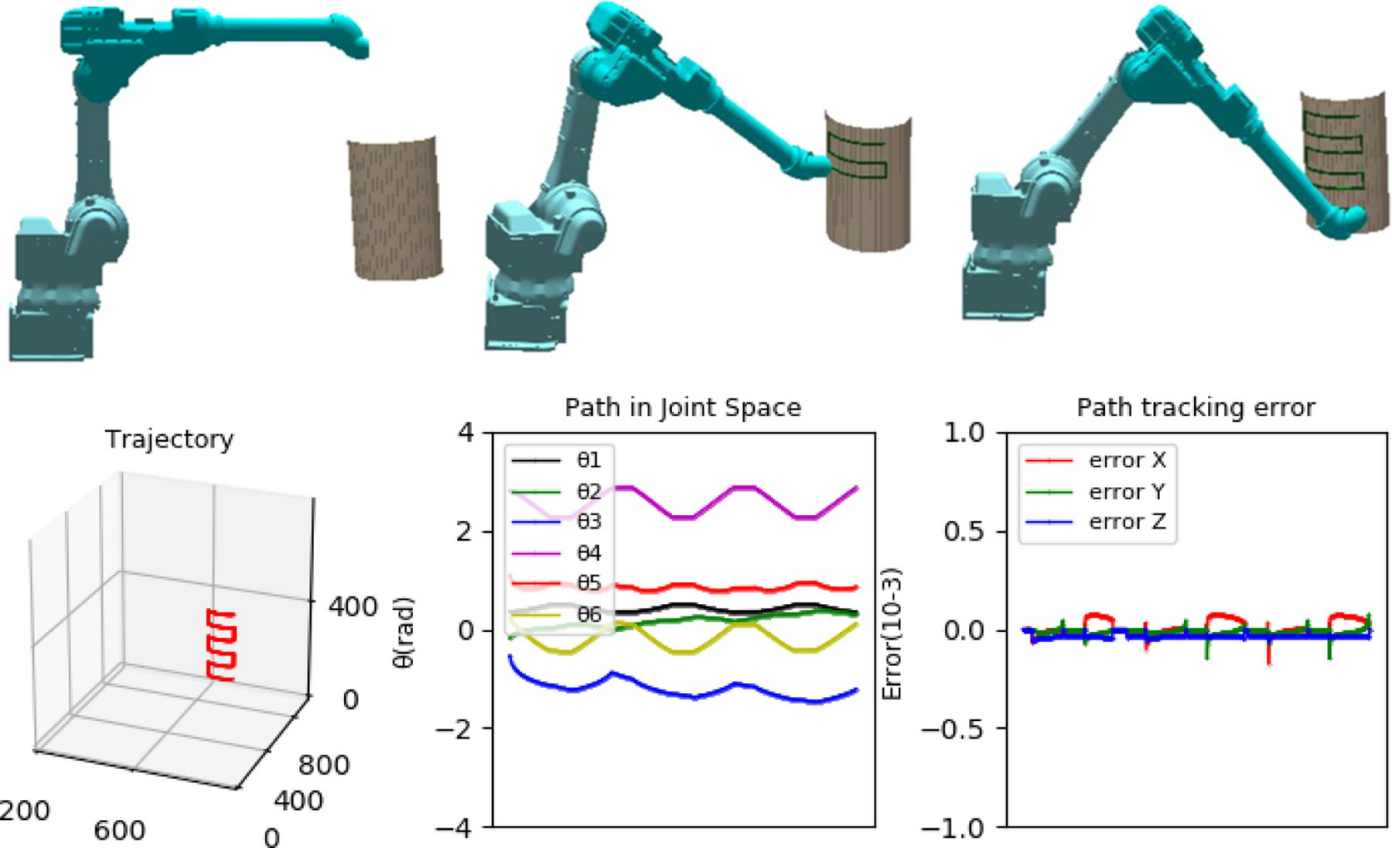
Simulation verification of robot.

A simulation platform was developed, based on Qt integrated opengl, QML and C ++ language. It was superior in cross-platform, high flexibility and high reusability, etc. Combined with the interpolation algorithm, a trajectory was planned. The algorithms in the paper were adopted, the robot model moved the planned trajectory on a curved surface in the simulation platform, and the collecting data were analyzed, as shown in Fig 10.

According to Fig 10, the planned trajectory of a robot model moving on a curved surface was accurate and the robot moved smoothly. The joint angle changed smoothly in the joint space, and the trajectory tracking errors were up to grade. The optimized method could ensure the stability and accuracy of the motion.

**Fig 11:**
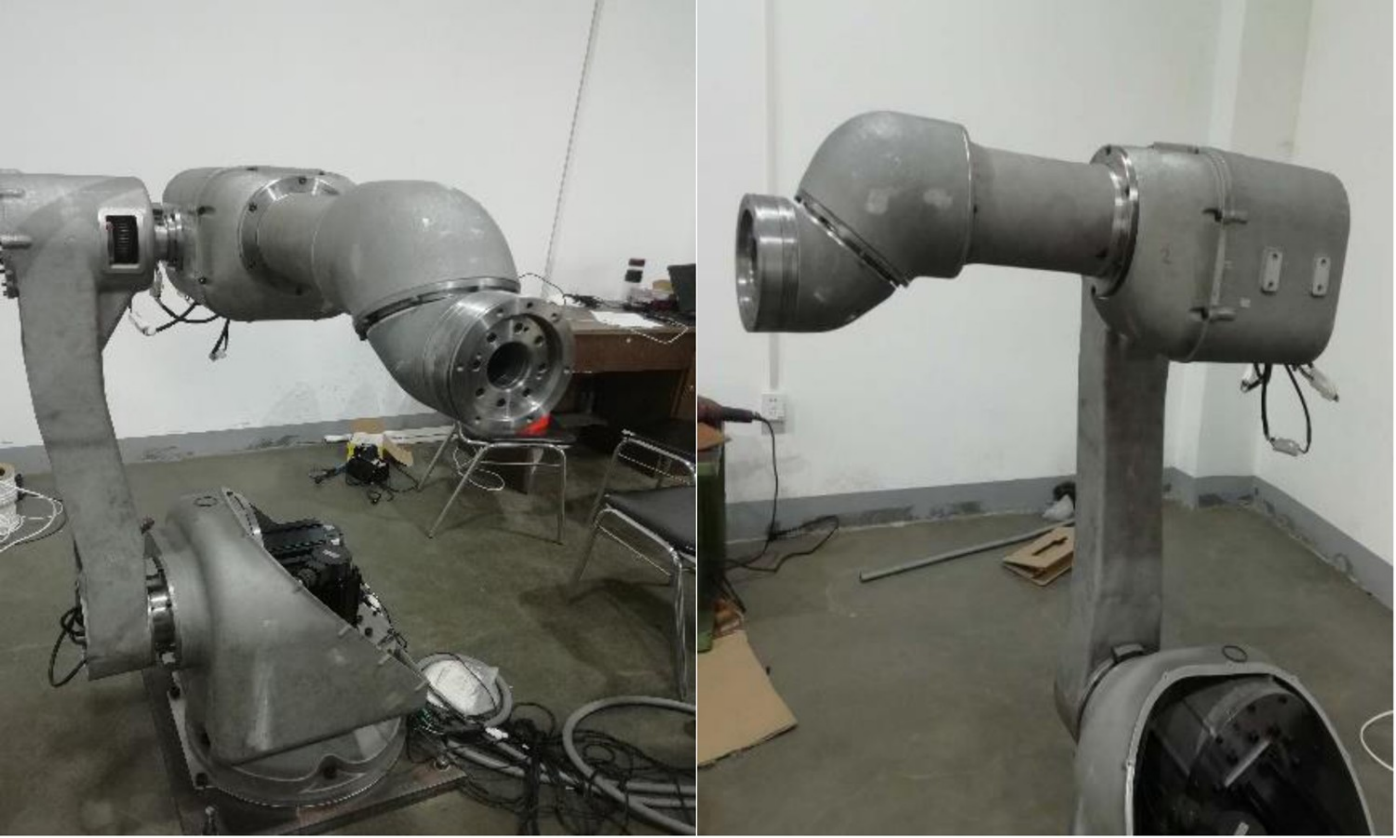
Experimental platform.

There was the experimental platform of oblique non-spherical wrist robot in the lab as shown in Fig 11. Same as the configuration of robot studied in the paper, the structure parameters were *a*_1_ = 0.135, *a*_2_ = 0.7, *a*_3_ = 0.1, *d*_4_ = 0.433, *d*_5_ = 0.115, *d*_6_ = 0.174, *β* = 60°. The two methods optimized in this paper were encoded and implemented on the DSP end of the controller, to control the motion of the robot at high speed and smoothly. The effectiveness of the methods were verified.

## Conclusions

For the orthogonal non-spherical wrist configuration, the simplest analytical solution was given in this paper. For the oblique non-spherical wrist configuration, elimination method and Gauss damped least squares method were selected to optimize the solution. For the elimination method, this paper mainly made three points optimization, gave the expression of equality simplification, deduced the expressions of final *A*, *B* and *C* matrix to improve the speed of solution, and gave the simplest expression of all joint variables to complement the integrity of elimination method. Elimination method was mainly time-consuming in solving eigenvalues and eigenvectors of *M* matrix. Increasing the speed of this step implies the total cost of the algorithm will be decreased. For the Gauss damped least squares method, the enhanced step length coefficient was proposed. By analyzing the influencing factors of the coefficient, the correlation between the coefficient and the initial iteration value was determined. The concept of optimal enhanced step length coefficient was introduced, and studied using the classification and regression methods of machine learning. By comparing the performance indicators, it was determined that the Decision Tree algorithm had better performance for the study of this problem. Follow-up research expects to obtain more accurate coefficients approaching the optimal enhanced step length coefficients through other methods, so that the number of iterations for each solution can be minimized.

## Supporting information

S1 Table Matlab code. (M)

S1 Appendix (DOCX)
