# Supplementary material for: The optimized algorithm based on machine learning for inverse kinematics of two painting robots with non-spherical wrist
Source: PLoS One. 2020 Apr 3;15(4):e0230790. doi: 10.1371/journal.pone.0230790 (PMC7122721; doi:10.1371/journal.pone.0230790)
Supplement: S1 Appendix — (DOCX) [file pone.0230790.s002.docx]

**Appendix**

$$A=\left[ \begin{aligned} m_{113}-m_{112},0,m_{133}-m_{132},m_{143}-m_{142},m_{153},m_{163}-m_{162}, \\ m_{213},m_{223}-m_{222},m_{233},m_{243}-m_{242},m_{253},0, \\ m_{313}-m_{312},m_{323},m_{333}-m_{332},m_{343},m_{353},m_{363}, \\ m_{413}-m_{412},m_{423}-m_{422},m_{433},0,m_{453}-m_{452},0, \\ m_{513}-m_{512},m_{523}-m_{522},m_{533}-m_{532},m_{543}-m_{542},0,-m_{562}, \\ m_{613}-m_{612},m_{623}-m_{622},m_{633}-m_{632},m_{643}-m_{642},0,m_{663}-m_{662}, \\ 0,0,0,m_{113}-m_{112},0,m_{133}-m_{132}, \\ 0,0,0,m_{213},m_{223}-m_{222},m_{233}, \\ 0,0,0,m_{313}-m_{312},m_{323},m_{333}-m_{332}, \\ 0,0,0,m_{413}-m_{412},m_{423}-m_{422},m_{433}, \\ 0,0,0,m_{513}-m_{512},m_{523}-m_{522},m_{533}-m_{532}, \\ 0,0,0,m_{613}-m_{612},m_{623}-m_{622},m_{633}-m_{632}, \end{aligned} \right.$$

$$\left. \begin{aligned} m_{173}-m_{172},0,m_{193}-m_{192},0,0,0 \\ m_{273},m_{283}-m_{282},m_{293},0,0,0 \\ m_{373}-m_{372},m_{383},m_{393}-m_{392},0,0,0 \\ m_{473}-m_{472},m_{483}-m_{482},m_{493},0,0,0 \\ m_{573}-m_{572},m_{583}-m_{582},m_{593}-m_{592},0,0,0 \\ m_{673}-m_{672},m_{683}-m_{682},m_{693}-m_{692},0,0,0 \\ m_{143}-m_{142},m_{153},m_{163}-m_{162},m_{173}-m_{172},0,m_{193}-m_{192} \\ m_{243}-m_{242},m_{253},0,m_{273},m_{283}-m_{282},m_{293} \\ m_{343},m_{353},m_{363},m_{373}-m_{372},m_{383},m_{393}-m_{392} \\ 0,m_{453}-m_{452},0,m_{473}-m_{472},m_{483}-m_{482},m_{493} \\ m_{543}-m_{542},0,-m_{562},m_{573}-m_{572},m_{583}-m_{582},m_{593}-m_{592} \\ m_{643}-m_{642},0,m_{663}-m_{662},m_{673}-m_{672},m_{683}-m_{682},m_{693}-m_{692} \end{aligned} \right]$$

$$B=\left[ \begin{aligned} 2m_{111},0,2m_{131},0,0,0,2m_{171},0,2m_{191},0,0,0 \\ 2m_{211},0,2m_{231},0,0,0,2m_{271},0,2m_{291}1,0,0,0 \\ 0,2m_{321},0,2m_{341},0,0,0,2m_{381},0,0,0,0 \\ 2m_{411},0,2m_{431},0,2m_{451},0,2m_{471},0,2m_{491},0,0,0 \\ 2m_{511},2m_{521},2m_{531},2m_{541},0,2m_{561},2m_{571},2m_{581},2m_{591},0,0,0 \\ 2m_{611},2m_{621},2m_{631},2m_{641},0,2m_{661},2m_{671},2m_{681},2m_{691},0,0,0 \\ 0,0,0,2m_{111},0,2m_{131},0,0,0,2m_{171},0,2m_{191} \\ 0,0,0,2m_{211},0,2m_{231},0,0,0,2m_{271},0,2m_{291} \\ 0,0,0,0,2m_{321},0,2m_{341},0,0,0,2m_{381},0 \\ 0,0,0,2m_{411},0,2m_{431},0,2m_{451},0,2m_{471},0,2m_{491} \\ 0,0,0,2m_{511},2m_{521},2m_{531},2m_{541},0,2m_{561},2m_{571},2m_{581},2m_{591} \\ 0,0,0,2m_{611},2m_{621},2m_{631},2m_{641},0,2m_{661},2m_{671},2m_{681},2m_{691} \end{aligned} \right]$$

$$C=\left[ \begin{aligned} m_{113}+m_{112},0,m_{133}+m_{132},m_{143}+m_{142},m_{153},m_{163}+m_{162}, \\ m_{213},m_{223}+m_{222},m_{233},m_{243}+m_{242},m_{253},0, \\ m_{313}+m_{312},m_{323},m_{333}+m_{332},m_{343},m_{353},m_{363}, \\ m_{413}+m_{412},m_{423}+m_{422},m_{433},0,m_{453}+m_{452},0, \\ m_{513}+m_{512},m_{523}+m_{522},m_{533}+m_{532},m_{543}+m_{542},0,m_{562}, \\ m_{613}+m_{612},m_{623}+m_{622},m_{633}+m_{632},m_{643}+m_{642},0,m_{663}+m_{662}, \\ 0,0,0,m_{113}+m_{112},0,m_{133}+m_{132}, \\ 0,0,0,m_{213},m_{223}+m_{222},m_{233}, \\ 0,0,0,m_{313}+m_{312},m_{323},m_{333}+m_{332}, \\ 0,0,0,m_{413}+m_{412},m_{423}+m_{422},m_{433}, \\ 0,0,0,m_{513}+m_{512},m_{523}+m_{522},m_{533}+m_{532}, \\ 0,0,0,m_{613}+m_{612},m_{623}+m_{622},m_{633}+m_{632}, \end{aligned} \right.$$

$$\left. \begin{aligned} m_{173}+m_{172},0,m_{193}+m_{192},0,0,0 \\ m_{273},m_{283}+m_{282},m_{293},0,0,0 \\ m_{373}+m_{372},m_{383},m_{393}+m_{392},0,0,0 \\ m_{473}+m_{472},m_{483}+m_{482},m_{493},0,0,0 \\ m_{573}+m_{572},m_{583}+m_{582},m_{593}+m_{592},0,0,0 \\ m_{673}+m_{672},m_{683}+m_{682},m_{693}+m_{692},0,0,0 \\ m_{143}+m_{142},m_{153},m_{163}+m_{162},m_{173}+m_{172},0,m_{193}+m_{192} \\ m_{243}+m_{242},m_{253},0,m_{273},m_{283}+m_{282},m_{293} \\ m_{343},m_{353},m_{363},m_{373}+m_{372},m_{383},m_{393}+m_{392} \\ 0,m_{453}+m_{452},0,m_{473}+m_{472},m_{483}+m_{482},m_{493} \\ m_{543}+m_{542},0,m_{562},m_{573}+m_{572},m_{583}+m_{582},m_{593}+m_{592} \\ m_{643}+m_{642},0,m_{663}+m_{662},m_{673}+m_{672},m_{683}+m_{682},m_{693}+m_{692} \end{aligned} \right]$$

$$f_{13}=\left. \left( d_{4}+c\beta d_{5} \right)^{2}-\left( p_{x}-d_{6}n_{x} \right)^{2}-\left( p_{y}-d_{6}n_{y} \right)^{2}-\left( pz-d_{6}n_{z} \right)^{2}-{a_{1}}^{2}+{a_{2}}^{2}+{a_{3}}^{2}+{d_{5}}^{2}{s\beta}^{2} \right.$$

$$f_{14}=2a_{1}\left( p_{x}-d_{6}n_{x} \right)\left( c\beta s\beta\left( p_{x}-d_{6}n_{x} \right)-d_{5}n_{x}s\beta\right)+ 2a_{1}\left( p_{y}-d_{6}n_{y} \right)\left( c\beta s\beta\left( p_{y}-d_{6}n_{y} \right)-d_{5}n_{y}s\beta\right)$$

$$f_{15}=c\beta s\beta(2a_{1}{(p_{x}-d_{6}n_{x})}^{2}+2a_{1}\left( p_{y}-d_{6}n_{y} \right)^{2})$$

$$f_{23}=(c\beta d_{5}-n_{x}p_{x}-n_{y}p_{y}-n_{z}p_{z}+{c\beta}^{2}d_{4}+d_{6}{n_{x}}^{2}+d_{6}{n_{y}}^{2}+d_{6}{n_{z}}^{2})(n_{x}p_{y}-n_{y}p_{x})$$

$$f_{24}=a_{1}n_{x}(c\beta s\beta(p_{x}-d_{6}n_{x})-d_{5}n_{x}s\beta)+ a_{1}n_{y}(c\beta s\beta(p_{y}-d_{6}n_{y})-d_{5}n_{y}s\beta)$$

$$f_{25}=c\beta s\beta(a_{1}n_{x}(p_{x}-d_{6}n_{x})+a_{1}n_{y}(p_{y}-d_{6}n_{y}))$$

$$f_{31}=(a_{3}{c\beta}^{2}-a_{1}n_{z})(n_{x}p_{y}-n_{y}p_{x})$$

$$f_{32}=(c\beta s\beta(p_{x}-d_{6}n_{x})-d_{5}n_{x}s\beta)(n_{x}p_{z}-n_{z}p_{x})+(c\beta s\beta(p_{y}-d_{6}n_{y})-d_{5}n_{y}s\beta)(n_{y}p_{z}-n_{z}p_{y})$$

$$f_{33}=c\beta s\beta((p_{x}-d_{6}n_{x})(n_{x}p_{z}-n_{z}p_{x})+(p_{y}-d_{6}n_{y})(n_{y}p_{z}-n_{z}p_{y}))$$

$$f_{41}=2d_{5}s\beta(c\beta d_{5}-n_{x}p_{x}-n_{y}p_{y}-n_{z}p_{z}+{c\beta}^{2}d_{4}+d_{6}{n_{x}}^{2}+d_{6}{n_{y}}^{2}+d_{6}{n_{z}}^{2})$$

$$f_{42}=2a_{1}n_{x}(p_{y}-d_{6}n_{y})-2a_{1}n_{y}(p_{x}-d_{6}n_{x})+2d_{5}s\beta d_{4}{s\beta}^{2}$$

$$f_{43}=2a_{1}n_{x}(p_{y}-d_{6}n_{y})-2a_{1}n_{y}(p_{x}-d_{6}n_{x})-2d_{5}s\beta d_{4}{s\beta}^{2}$$

$$m_{113}=\left( n_{x}p_{y}-n_{y}p_{x} \right)f_{13}+f_{14}+f_{15}$$

$$m_{133}=\left( n_{x}p_{y}-n_{y}p_{x} \right)f_{13}+f_{14}-f_{15}$$

$$m_{153}=-4s\beta(2a_{1}{(p_{x}-d_{6}n_{x})}^{2}+2a_{1}{(p_{y}-d_{6}n_{y})}^{2})$$

$$m_{173}=\left( n_{x}p_{y}-n_{y}p_{x} \right)f_{13}-f_{14}-f_{15}$$

$$m_{193}=\left( n_{x}p_{y}-n_{y}p_{x} \right)f_{13}-f_{14}+f_{15}$$

$$m_{213}=f_{23}+f_{24}+f_{25}-d_{4}{s\beta}^{2}(n_{x}p_{y}-n_{y}p_{x})$$

$$m_{233}=f_{23}+d_{4}{s\beta}^{2}(n_{x}p_{y}-n_{y}p_{x})+f_{24}-f_{25}$$

$$m_{253}=-4s\beta(a_{1}n_{x}(p_{x}-d_{6}n_{x})+a_{1}n_{y}(p_{y}-d_{6}n_{y}))$$

$$m_{273}=f_{23}-d_{4}{s\beta}^{2}(n_{x}p_{y}-n_{y}p_{x})-f_{24}-f_{25}$$

$$m_{293}=f_{23}+d_{4}{s\beta}^{2}(n_{x}p_{y}- n_{y}p_{x})-f_{24}+f_{25}$$

$$m_{313}=f_{31}-f_{32}-f_{33}-a_{3}{s\beta}^{2}(n_{x}p_{y}-n_{y}p_{x})$$

$$m_{333}=f_{31}-f_{32}+f_{33}+a_{3}{s\beta}^{2}(n_{x}p_{y}-n_{y}p_{x})$$

$$m_{343}=\left( - 2d_{5}s\beta-4c\beta d_{4}s\beta\right)(n_{x}p_{y}-n_{y}p_{x})$$

$$m_{353}=4s\beta((p_{x}-d_{6}n_{x})(n_{x}p_{z}-n_{z}p_{x})+ (p_{y}-d_{6}n_{y})(n_{y}p_{z}-n_{z}p_{y}))$$

$$m_{363}=2d_{5}s\beta(n_{x}p_{y}- n_{y}p_{x})$$

$$m_{373}=f_{31}+f_{32}+f_{33}-a_{3}{s\beta}^{2}(n_{x}p_{y}-n_{y}p_{x})$$

$$m_{393}=f_{31}+f_{32}-f_{33}+a_{3}{s\beta}^{2}(n_{x}p_{y}-n_{y}p_{x})$$

$$m_{413}=f_{42}-f_{41}+2c\beta s\beta f_{13}$$

$$m_{433}=f_{43}-f_{41}$$

$$m_{451}=-4s\beta(2a_{2}d_{4}+2a_{2}c\beta d_{5})$$

$$m_{452}=-8s\beta a_{2}a_{3}$$

$$m_{453}=-4s\beta f_{13}$$

$$m_{473}=f_{43}+f_{41}-2c\beta s\beta f_{13}$$

$$m_{493}=f_{42}+f_{41}$$

$$m_{111}= m_{131}= m_{171}= m_{191}=(2a_{2}d_{4}+2a_{2}c\beta d_{5})(n_{x}p_{y}-n_{y}p_{x})$$

$$m_{112}= m_{132}= m_{172}= m_{192}=2a_{2}a_{3}(n_{x}p_{y}- n_{y}p_{x})$$

$$m_{142}=m_{162}=4a_{2}d_{5}s\beta(n_{x}p_{y}-n_{y}p_{x})$$

$$m_{143}=m_{163}=4a_{3}d_{5}s\beta(n_{x}p_{y}-n_{y}p_{x})$$

$$m_{211}=m_{271}=m_{312}=m_{372}=(-a_{2}{s\beta}^{2}+a_{2}{c\beta}^{2})(n_{x}p_{y}-n_{y}p_{x})$$

$$m_{231}= m_{291}= m_{332}= m_{392}=(a_{2}{s\beta}^{2}+a_{2}{c\beta}^{2})(n_{x}p_{y}-n_{y}p_{x})$$

$$m_{223}= m_{243}=- m_{283}=2a_{3}s\beta(n_{x}p_{y}-n_{y}p_{x})$$

$$m_{222}= m_{242}=-m_{282}=- m_{321}= -m_{341}= m_{381}=2a_{2}s\beta(n_{x}p_{y}-n_{y}p_{x})$$

$$m_{323}=- m_{383}=(- 2d_{4}s\beta-2c\beta d_{5}s\beta)(n_{x}p_{y}-n_{y}p_{x})$$

$$m_{431}=-m_{491}= -2d_{5}s\beta a_{2}{s\beta}^{2}-2d_{5}s\beta a_{2}{c\beta}^{2}$$

$$m_{423}=m_{483}=-4d_{5}a_{3}{s\beta}^{2}$$

$$m_{422}= m_{482}=-4d_{5}a_{2}{s\beta}^{2}$$

$$m_{412}=- m_{472}=4c\beta s\beta a_{2}a_{3}$$

$$m_{411}=-m_{471}=2d_{5}a_{2}{s\beta}^{3}-2d_{5}s\beta a_{2}{c\beta}^{2}+4c\beta s\beta a_{2}d_{4}+ 4{c\beta}^{2}s\beta a_{2}d_{5}$$

$$m_{512}=m_{572}=-m_{611}=-m_{671}=p_{z}s\beta^{2}+d_{4}n_{z}-{c\beta}^{2}p_{z}+{c\beta}^{2}d_{6}n_{z}-d_{6}n_{z}{s\beta}^{2}+c\beta d_{5}n_{z}-a_{3}{c\beta}^{2}d_{4}/a_{1}-a_{3}{c\beta}^{3}d_{5}/a_{1}-a_{3}d_{6}{n_{x}}^{2}/a_{1}-a_{3}d_{6}{n_{y}}^{2}/a_{1}-a_{3}d_{6}{n_{z}}^{2}/a_{1}+a_{3}d_{4}{s\beta}^{2}/a_{1}+a_{3}n_{x}p_{x}/a_{1}+a_{3}n_{y}p_{y}/a_{1}+a_{3}n_{z}p_{z}/a_{1}-a_{3}c\beta d_{5}{s\beta}^{2}/a_{1}$$

$$m_{513}=m_{573}=a_{2}n_{x}p_{x}/a_{1}+a_{2}n_{y}p_{y}/a_{1}+a_{2}n_{z}p_{z}/a_{1}-a_{2}{c\beta}^{2}d_{4}/a_{1}-a_{2}{c\beta}^{3}d_{5}/a_{1}-a_{2}d_{6}{n_{x}}^{2}/a_{1}-a_{2}d_{6}{n_{y}}^{2}/a_{1}-a_{2}d_{6}{n_{z}}^{2}/a_{1}+a_{2}d_{4}{s\beta}^{2}/a_{1}-a_{2}c\beta d_{5}{s\beta}^{2}/a_{1}$$

$$m_{521}=-m_{581}=m_{622}=-m_{682}=2p_{z}s\beta-2d_{6}n_{z}s\beta-2a_{3}d_{4}s\beta/a_{1}-2a_{3}c\beta d_{5}s\beta/a_{1}$$

$$m_{511}=m_{571}=-a_{1}{s\beta}^{2}/2-a_{3}n_{z}+a_{1}{c\beta}^{2}/2-{a_{2}}^{2}{c\beta}^{2}/(2a_{1})+{a_{3}}^{2}{c\beta}^{2}/(2a_{1})-{c\beta}^{2}{d_{4}}^{2}/(2a_{1})-{c\beta}^{4}{d_{5}}^{2}/(2a_{1})-{c\beta}^{2}{p_{x}}^{2}/(2a_{1})-{c\beta}^{2}{p_{y}}^{2}/(2a_{1})-{c\beta}^{2}{p_{z}}^{2}/(2a_{1})+{a_{2}}^{2}{s\beta}^{2}/(2a_{1})-{a_{3}}^{2}{s\beta}^{2}/(2a_{1})+{d_{4}}^{2}{s\beta}^{2}/(2a_{1})-{d_{5}}^{2}{s\beta}^{4}/(2a_{1})+{p_{x}}^{2}{s\beta}^{2}/(2a_{1})+{p_{y}}^{2}{s\beta}^{2}/(2a_{1})+{p_{z}}^{2}{s\beta}^{2}/(2a_{1})-{c\beta}^{3}d_{4}d_{5}/a_{1}-d_{4}d_{6}{n_{x}}^{2}/a_{1}-d_{4}d_{6}{n_{y}}^{2}/a_{1}-d_{4}d_{6}{n_{z}}^{2}/a_{1}+d_{4}n_{x}p_{x}/a_{1}+d_{4}n_{y}p_{y}/a_{1}+d_{4}n_{z}p_{z}/a_{1}-{c\beta}^{2}{d_{6}}^{2}{n_{x}}^{2}/(2a_{1})-{c\beta}^{2}{d_{6}}^{2}{n_{y}}^{2}/(2a_{1})-{c\beta}^{2}{d_{6}}^{2}{n_{z}}^{2}/(2a_{1})-{c\beta}^{2}{d_{5}}^{2}{s\beta}^{2}/a_{1}+{d_{6}}^{2}{n_{x}}^{2}{s\beta}^{2}/(2a_{1})+{d_{6}}^{2}{n_{y}}^{2}{s\beta}^{2}/(2a_{1})+{d_{6}}^{2}{n_{z}}^{2}{s\beta}^{2}/(2a_{1})+c\beta d_{5}n_{x}p_{x}/a_{1}+c\beta d_{5}n_{y}p_{y}/a_{1}+c\beta d_{5}n_{z}p_{z}/a_{1}-c\beta d_{5}d_{6}{n_{x}}^{2}/a_{1}-c\beta d_{5}d_{6}{n_{y}}^{2}/a_{1}-c\beta d_{5}d_{6}{n_{z}}^{2}/a_{1}-c\beta d_{4}d_{5}{s\beta}^{2}/a_{1}+{c\beta}^{2}d_{6}n_{x}p_{x}/a_{1}+{c\beta}^{2}d_{6}n_{y}p_{y}/a_{1}+{c\beta}^{2}d_{6}n_{z}p_{z}/a_{1}-d_{6}n_{x}p_{x}{s\beta}^{2}/a_{1}-d_{6}n_{y}p_{y}{s\beta}^{2}/a_{1}-d_{6}n_{z}p_{z}{s\beta}^{2}/a_{1}$$

$$m_{522}=-m_{582}=a_{1}s\beta+{d_{5}}^{2}{s\beta}^{3}/a_{1}-{a_{2}}^{2}s\beta/a_{1}-{a_{3}}^{2}s\beta/a_{1}+{d_{4}}^{2}s\beta/a_{1}-{p_{x}}^{2}s\beta/a_{1}-{p_{y}}^{2}s\beta/a_{1}-{p_{z}}^{2}s\beta/a_{1}+{c\beta}^{2}{d_{5}}^{2}s\beta/a_{1}-{d_{6}}^{2}{n_{x}}^{2}s\beta/a_{1}-{d_{6}}^{2}{n_{y}}^{2}s\beta/a_{1}-{d_{6}}^{2}{n_{z}}^{2}s\beta/a_{1}+2c\beta d_{4}d_{5}s\beta/a_{1}+2d_{6}n_{x}p_{x}s\beta/a_{1}+2d_{6}n_{y}p_{y}s\beta/a_{1}+2d_{6}n_{z}p_{z}s\beta/a_{1}$$

$$m_{523}=m_{543}=-m_{583}=-2a_{2}a_{3}s\beta/a_{1}$$

$$m_{531}=m_{591}=a_{1}{s\beta}^{2}/2-a_{3}n_{z}+a_{1}{c\beta}^{2}/2-{a_{2}}^{2}{c\beta}^{2}/(2a_{1})+{a_{3}}^{2}{c\beta}^{2}/(2a_{1})-{c\beta}^{2}{d_{4}}^{2}/(2a_{1})-{c\beta}^{4}{d_{5}}^{2}/(2a_{1})-{c\beta}^{2}{p_{x}}^{2}/(2a_{1})-{c\beta}^{2}{p_{y}}^{2}/(2a_{1})-{c\beta}^{2}{p_{z}}^{2}/(2a_{1})-{a_{2}}^{2}{s\beta}^{2}/(2a_{1})+{a_{3}}^{2}{s\beta}^{2}/(2a_{1})-{d_{4}}^{2}{s\beta}^{2}/(2a_{1})+{d_{5}}^{2}{s\beta}^{4}/(2a_{1})-{p_{x}}^{2}{s\beta}^{2}/(2a_{1})-{p_{y}}^{2}{s\beta}^{2}/(2a_{1})-{p_{z}}^{2}{s\beta}^{2}/(2a_{1})-{c\beta}^{3}d_{4}d_{5}/a_{1}-d_{4}d_{6}{n_{x}}^{2}/a_{1}-d_{4}d_{6}{n_{y}}^{2}/a_{1}-d_{4}d_{6}{n_{z}}^{2}/a_{1}+d_{4}n_{x}p_{x}/a_{1}+d_{4}n_{y}p_{y}/a_{1}+d_{4}n_{z}p_{z}/a_{1}-{c\beta}^{2}{d_{6}}^{2}{n_{x}}^{2}/(2a_{1})-{c\beta}^{2}{d_{6}}^{2}{n_{y}}^{2}/(2a_{1})-{c\beta}^{2}d_{6}{n_{z}}^{2}/(2a_{1})-{d_{6}}^{2}{n_{x}}^{2}{s\beta}^{2}/(2a_{1})-{d_{6}}^{2}{n_{y}}^{2}{s\beta}^{2}/(2a_{1})-{d_{6}}^{2}{n_{z}}^{2}{s\beta}^{2}/(2a_{1})+c\beta d_{5}n_{x}p_{x}/a_{1}+c\beta d_{5}n_{y}p_{y}/a_{1}+c\beta d_{5}n_{z}p_{z}/a_{1}-c\beta d_{5}d_{6}{n_{x}}^{2}/a_{1}-c\beta d_{5}d_{6}{n_{y}}^{2}/a_{1}-c\beta d_{5}d_{6}{n_{z}}^{2}/a_{1}-c\beta d_{4}d_{5}{s\beta}^{2}/a_{1}+{c\beta}^{2}d_{6}n_{x}p_{x}/a_{1}+{c\beta}^{2}d_{6}n_{y}p_{y}/a_{1}+{c\beta}^{2}d_{6}n_{z}p_{z}/a_{1}+d_{6}n_{x}p_{x}{s\beta}^{2}/a_{1}+d_{6}n_{y}p_{y}{s\beta}^{2}/a_{1}+d_{6}n_{z}p_{z}{s\beta}^{2}/a_{1}$$

$$m_{532}=m_{592}=-m_{631}=-m_{691}=-p_{z}{s\beta}^{2}+d_{4}n_{z}-{c\beta}^{2}p_{z}+{c\beta}^{2}d_{6}n_{z}+d_{6}n_{z}{s\beta}^{2}+c\beta d_{5}n_{z}-a_{3}{c\beta}^{2}d_{4}/a_{1}-a_{3}{c\beta}^{3}d_{5}/a_{1}-a_{3}d_{6}{n_{x}}^{2}/a_{1}-a_{3}d_{6}{n_{y}}^{2}/a_{1}-a_{3}d_{6}{n_{z}}^{2}/a_{1}-a_{3}d_{4}{s\beta}^{2}/a_{1}+a_{3}n_{x}p_{x}/a_{1}+a_{3}n_{y}p_{y}/a_{1}+a_{3}n_{z}p_{z}/a_{1}-a_{3}c\beta d_{5}{s\beta}^{2}/a_{1}$$

$$m_{533}=m_{593}=a_{2}n_{x}p_{x}/a_{1}+a_{2}n_{y}p_{y}/a_{1}+a_{2}n_{z}p_{z}/a_{1}-a_{2}{c\beta}^{2}d_{4}/a_{1}-a_{2}{c\beta}^{3}d_{5}/a_{1}-a_{2}d_{6}{n_{x}}^{2}/a_{1}-a_{2}d_{6}{n_{y}}^{2}/a_{1}-a_{2}d_{6}{n_{z}}^{2}/a_{1}-a_{2}d_{4}{s\beta}^{2}/a_{1}-a_{2}c\beta d_{5}{s\beta}^{2}/a_{1}$$

$$m_{541}=m_{642}=4c\beta p_{z}s\beta-2d_{5}n_{z}s\beta-2a_{3}d_{5}{s\beta}^{3}/a_{1}-4c\beta d_{6}n_{z}s\beta-4a_{3}c\beta d_{4}s\beta/a_{1}-2a_{3}{c\beta}^{2}d_{5}s\beta/a_{1}$$

$$m_{542}=2a_{1}c\beta s\beta-2{a_{2}}^{2}c\beta s\beta/a_{1}-2{a_{3}}^{2}c\beta s\beta/a_{1}+2c\beta{d_{4}}^{2}s\beta/a_{1}+2d_{4}d_{5}{s\beta}^{3}/a_{1}-2c\beta{p_{x}}^{2}s\beta/a_{1}-2c\beta{p_{y}}^{2}s\beta/a_{1}-2c\beta{p_{z}}^{2}s\beta/a_{1}+2d_{5}n_{x}p_{x}s\beta/a_{1}+2d_{5}n_{y}p_{y}s\beta/a_{1}+2d_{5}n_{z}p_{z}s\beta/a_{1}+2{c\beta}^{2}d_{4}d_{5}s\beta/a_{1}-2d_{5}d_{6}{n_{x}}^{2}s\beta/a_{1}-2d_{5}d_{6}{n_{y}}^{2}s\beta/a_{1}-2d_{5}d_{6}{n_{z}}^{2}s\beta/a_{1}-2c\beta{d_{6}}^{2}{n_{x}}^{2}s\beta/a_{1}-2c\beta{d_{6}}^{2}{n_{y}}^{2}s\beta/a_{1}-2c\beta{d_{6}}^{2}{n_{z}}^{2}s\beta/a_{1}+4c\beta d_{6}n_{x}p_{x}s\beta/a_{1}+4c\beta d_{6}n_{y}p_{y}s\beta/a_{1}+4c\beta d_{6}n_{z}p_{z}s\beta/a_{1}$$

$$m_{561}=m_{662}=2a_{3}d_{5}{s\beta}^{3}/a_{1}-2d_{5}n_{z}s\beta+2a_{3}{c\beta}^{2}d_{5}s\beta/a_{1}$$

$$m_{661}=-m_{562}=2c\beta{d_{5}}^{2}s\beta/a_{1}+2d_{4}d_{5}s\beta/a_{1}-2d_{5}n_{x}p_{x}s\beta/a_{1}-2d_{5}n_{y}p_{y}s\beta/a_{1}-2d_{5}n_{z}p_{z}s\beta/a_{1}+2d_{5}d_{6}{n_{x}}^{2}s\beta/a_{1}+2d_{5}d_{6}{n_{y}}^{2}s\beta/a_{1}+2d_{5}d_{6}{n_{z}}^{2}s\beta/a_{1}$$

$$m_{672}=m_{612}=-a_{3}n_{z}+a_{1}{c\beta}^{2}/2-a_{1}{s\beta}^{2}/2+{a_{2}}^{2}{c\beta}^{2}/(2a_{1})+{a_{3}}^{2}{c\beta}^{2}/(2a_{1})-{c\beta}^{2}{d_{4}}^{2}/(2a_{1})-{c\beta}^{4}{d_{5}}^{2}/(2a_{1})-{c\beta}^{2}{p_{x}}^{2}/(2a_{1})-{c\beta}^{2}{p_{y}}^{2}/(2a_{1})-{c\beta}^{2}{p_{z}}^{2}/(2a_{1})-{a_{2}}^{2}{s\beta}^{2}/(2a_{1})-{a_{3}}^{2}{s\beta}^{2}/(2a_{1})+{d_{4}}^{2}{s\beta}^{2}/(2a_{1})-{d_{5}}^{2}{s\beta}^{2}/(2a_{1})+{p_{x}}^{2}{s\beta}^{2}/(2a_{1})+{p_{y}}^{2}{s\beta}^{2}/(2a_{1})+{p_{z}}^{2}{s\beta}^{2}/(2a_{1})-{c\beta}^{3}d_{4}d_{5}/a_{1}-d_{4}d_{6}{n_{x}}^{2}/a_{1}-d_{4}d_{6}{n_{y}}^{2}/a_{1}-d_{4}d_{6}{n_{z}}^{2}/a_{1}+d_{4}n_{x}p_{x}/a_{1}+d_{4}n_{y}p_{y}/a_{1}+d_{4}n_{z}p_{z}/a_{1}-{c\beta}^{2}{d_{6}}^{2}{n_{x}}^{2}/(2a_{1})-{c\beta}^{2}{d_{6}}^{2}{n_{y}}^{2}/(2a_{1})-{c\beta}^{2}{d_{6}}^{2}{n_{z}}^{2}/(2a_{1})-{c\beta}^{2}{d_{5}}^{2}{s\beta}^{2}/(2a_{1})+{d_{6}}^{2}{n_{x}}^{2}{s\beta}^{2}/(2a_{1})+{d_{6}}^{2}{n_{y}}^{2}{s\beta}^{2}/(2a_{1})+{d_{6}}^{2}{n_{z}}^{2}{s\beta}^{2}/(2a_{1})+c\beta d_{5}n_{x}p_{x}/a_{1}+c\beta d_{5}n_{y}p_{y}/a_{1}+c\beta d_{5}n_{z}p_{z}/a_{1}-c\beta d_{5}d_{6}{n_{x}}^{2}/a_{1}-c\beta d_{5}d_{6}{n_{y}}^{2}/a_{1}-c\beta d_{5}d_{6}{n_{z}}^{2}/a_{1}-c\beta d_{4}d_{5}{s\beta}^{2}/a_{1}+{c\beta}^{2}d_{6}n_{x}p_{x}/a_{1}+{c\beta}^{2}d_{6}n_{y}p_{y}/a_{1}+{c\beta}^{2}d_{6}n_{z}p_{z}/a_{1}-d_{6}n_{x}p_{x}{s\beta}^{2}/a_{1}-d_{6}n_{y}p_{y}{s\beta}^{2}/a_{1}-d_{6}n_{z}p_{z}{s\beta}^{2}/a_{1}$$

$$m_{613}=m_{673}=-a_{2}n_{z}+a_{2}a_{3}{c\beta}^{2}/a_{1}-a_{2}a_{3}{s\beta}^{2}/a_{1}$$

$$m_{621}=-m_{681}=-a_{1}s\beta-{a_{2}}^{2}s\beta/a_{1}+{a_{3}}^{2}s\beta/a_{1}-{d_{4}}^{2}s\beta/a_{1}-{d_{5}}^{2}s\beta/a_{1}+{p_{x}}^{2}s\beta/a_{1}+{p_{y}}^{2}s\beta/a_{1}+{p_{z}}^{2}s\beta/a_{1}+{d_{6}}^{2}{n_{x}}^{2}s\beta/a_{1}+{d_{6}}^{2}{n_{y}}^{2}s\beta/a_{1}+{d_{6}}^{2}{n_{z}}^{2}s\beta/a_{1}-2c\beta d_{4}d_{5}s\beta/a_{1}-2d_{6}n_{x}p_{x}s\beta/a_{1}-2d_{6}n_{y}p_{y}s\beta/a_{1}-2d_{6}n_{z}p_{z}s\beta/a_{1}$$

$$m_{623}=-m_{683}=-2a_{2}d_{4}s\beta/a_{1}-2a_{2}c\beta d_{5}s\beta/a_{1}$$

$$m_{632}=m_{692}=-a_{3}n_{z}+a_{1}{c\beta}^{2}/2+a_{1}{s\beta}^{2}/2+{a_{2}}^{2}{c\beta}^{2}/(2a_{1})+{a_{3}}^{2}{c\beta}^{2}/(2a_{1})-{c\beta}^{2}{d_{4}}^{2}/(2a_{1})-{c\beta}^{4}{d_{5}}^{2}/(2a_{1})-{c\beta}^{2}{p_{x}}^{2}/(2a_{1})-{c\beta}^{2}{p_{y}}^{2}/(2a_{1})-{c\beta}^{2}{p_{z}}^{2}/(2a_{1})+{a_{2}}^{2}{s\beta}^{2}/(2a_{1})+{a_{3}}^{2}{s\beta}^{2}/(2a_{1})-{d_{4}}^{2}{s\beta}^{2}/(2a_{1})+{d_{5}}^{2}{s\beta}^{2}/(2a_{1})-{p_{x}}^{2}{s\beta}^{2}/(2a_{1})-{p_{y}}^{2}{s\beta}^{2}/(2a_{1})-{p_{z}}^{2}{s\beta}^{2}/(2a_{1})-{c\beta}^{3}d_{4}d_{5}/a_{1}-d_{4}d_{6}{n_{x}}^{2}/a_{1}-d_{4}d_{6}{n_{y}}^{2}/a_{1}-d_{4}d_{6}{n_{z}}^{2}/a_{1}+d_{4}n_{x}p_{x}/a_{1}+d_{4}n_{y}p_{y}/a_{1}+d_{4}n_{z}p_{z}/a_{1}-{c\beta}^{2}{d_{6}}^{2}{n_{x}}^{2}/(2a_{1})-{c\beta}^{2}{d_{6}}^{2}{n_{y}}^{2}/(2a_{1})-{c\beta}^{2}{d_{6}}^{2}{n_{z}}^{2}/(2a_{1})-{c\beta}^{2}{d_{5}}^{2}{s\beta}^{2}/(2a_{1})-{d_{6}}^{2}{n_{x}}^{2}{s\beta}^{2}/(2a_{1})-{d_{6}}^{2}{n_{y}}^{2}{s\beta}^{2}/(2a_{1})-{d_{6}}^{2}{n_{z}}^{2}{s\beta}^{2}/(2a_{1})+c\beta d_{5}n_{x}p_{x}/a_{1}+c\beta d_{5}n_{y}p_{y}/a_{1}+c\beta d_{5}n_{z}p_{z}/a_{1}-c\beta d_{5}d_{6}{n_{x}}^{2}/a_{1}-c\beta d_{5}d_{6}{n_{y}}^{2}/a_{1}-c\beta d_{5}d_{6}{n_{z}}^{2}/a_{1}-c\beta d_{4}d_{5}{s\beta}^{2}/a_{1}+{c\beta}^{2}d_{6}n_{x}p_{x}/a_{1}+{c\beta}^{2}d_{6}n_{y}p_{y}/a_{1}+{c\beta}^{2}d_{6}n_{z}p_{z}/a_{1}+d_{6}n_{x}p_{x}{s\beta}^{2}/a_{1}+d_{6}n_{y}p_{y}{s\beta}^{2}/a_{1}+d_{6}n_{z}p_{z}{s\beta}^{2}/a_{1}$$

$$m_{633}=m_{693}=-a_{2}n_{z}+a_{2}a_{3}{c\beta}^{2}/a_{1}+a_{2}a_{3}{s\beta}^{2}/a_{1}$$

$$m_{641}=-2a_{1}c\beta s\beta-2{a_{2}}^{2}c\beta s\beta/a_{1}+2{a_{3}}^{2}c\beta s\beta/a_{1}-2c\beta{d_{4}}^{2}s\beta/a_{1}+2c\beta{p_{x}}^{2}s\beta/a_{1}+2c\beta{p_{y}}^{2}s\beta/a_{1}+2c\beta{p_{z}}^{2}s\beta/a_{1}-2d_{4}d_{5}s\beta/a_{1}-2d_{5}n_{x}p_{x}s\beta/a_{1}-2d_{5}n_{y}p_{y}s\beta/a_{1}-2d_{5}n_{z}p_{z}s\beta/a1+2d_{5}d_{6}{n_{x}}^{2}s\beta/a_{1}+2d_{5}d_{6}{n_{y}}^{2}s\beta/a_{1}+2d_{5}d_{6}{n_{z}}^{2}s\beta/a_{1}+2c\beta{d_{6}}^{2}{n_{x}}^{2}s\beta/a_{1}+2c\beta{d_{6}}^{2}{n_{y}}^{2}s\beta/a_{1}+2c\beta{d_{6}}^{2}{n_{z}}^{2}s\beta/a_{1}-4c\beta d_{6}n_{x}p_{x}s\beta/a_{1}-4c\beta d_{6}n_{y}p_{y}s\beta/a_{1}-4c\beta d_{6}n_{z}p_{z}s\beta/a_{1}$$

$$m_{643}=-2a_{2}d_{5}s\beta/a_{1}-4a_{2}c\beta d_{4}s\beta/a_{1}$$

$$m_{663}=2a_{2}d_{5}s\beta/a_{1}$$
